# A Role for Calcium-Permeable AMPA Receptors in Synaptic Plasticity and Learning

**DOI:** 10.1371/journal.pone.0012818

**Published:** 2010-09-29

**Authors:** Brian J. Wiltgen, Gordon A. Royle, Erin E. Gray, Andrea Abdipranoto, Nopporn Thangthaeng, Nate Jacobs, Faysal Saab, Susumu Tonegawa, Stephen F. Heinemann, Thomas J. O'Dell, Michael S. Fanselow, Bryce Vissel

**Affiliations:** 1 Department of Psychology, University of Virginia, Charlottesville, Virginia, United States of America; 2 Department of Psychology and the Brain Research Institute, University of California Los Angeles, Los Angeles, California, United States of America; 3 Neural Plasticity and Regeneration Research Group, Neuroscience Research Program, Garvan Institute of Medical Research, Sydney, New South Wales, Australia; 4 Interdepartmental Ph.D. Program for Neuroscience, University of California Los Angeles, Los Angeles, California, United States of America; 5 Picower Institute for Learning and Memory, Massachusetts Institute of Technology (MIT), Boston, Massachusetts, United States of America; 6 Department of Physiology, David Geffen School of Medicine at UCLA, Los Angeles, California, United States of America; 7 Molecular Neurobiology Department, Salk Institute, La Jolla, California, United States of America; Duke University, United States of America

## Abstract

A central concept in the field of learning and memory is that NMDARs are essential for synaptic plasticity and memory formation. Surprisingly then, multiple studies have found that behavioral experience can reduce or eliminate the contribution of these receptors to learning. The cellular mechanisms that mediate learning in the absence of NMDAR activation are currently unknown. To address this issue, we examined the contribution of Ca^2+^-permeable AMPARs to learning and plasticity in the hippocampus. Mutant mice were engineered with a conditional genetic deletion of GluR2 in the CA1 region of the hippocampus (GluR2-cKO mice). Electrophysiology experiments in these animals revealed a novel form of long-term potentiation (LTP) that was independent of NMDARs and mediated by GluR2-lacking Ca^2+^-permeable AMPARs. Behavioral analyses found that GluR2-cKO mice were impaired on multiple hippocampus-dependent learning tasks that required NMDAR activation. This suggests that AMPAR-mediated LTP interferes with NMDAR-dependent plasticity. In contrast, NMDAR-independent learning was normal in knockout mice and required the activation of Ca^2+^-permeable AMPARs. These results suggest that GluR2-lacking AMPARs play a functional and previously unidentified role in learning; they appear to mediate changes in synaptic strength that occur after plasticity has been established by NMDARs.

## Introduction

N-methyl-D-aspartate receptors (NMDARs) are necessary for most forms of synaptic plasticity in the CA1 region of the hippocampus [1]. Activation of these receptors is also essential for spatial and contextual learning [2], [3], [4]. Once learning has been established, however, new memories can often be formed without NMDARs. This surprising discovery was first described in the now-classic ‘upstairs/downstairs’ watermaze studies [5], [6]. In these experiments, rats were trained in a maze located on the lower floor of a laboratory and were subsequently able to acquire spatial information about a second upstairs maze even in the presence of the NMDAR antagonist APV. The same effect has been observed using contextual fear conditioning [7], [8]. These results imply that the NMDAR is not required for all forms of hippocampus-dependent learning and suggest that alternative plasticity mechanisms become available following recent behavioral experience.

A similar phenomenon occurs in the rodent barrel cortex. In this region, single whisker experience (SWE) induces NMDAR-dependent LTP at layer 4-2/3 synapses. Following this experience, however, subsequent increases in synaptic strength are independent of NMDAR activation [9]. Therefore, similar to spatial and context learning in the hippocampus, SWE produces NMDAR-independent plasticity in the barrel cortex. The activation of NMDARs in both of these brain regions has been shown to facilitate the delivery of GluR2-lacking (Ca^2+^-permeable) AMPARs to the synapse [9], [10], [11], [12], [13], but see [14], [15]. A number of recent studies have also found that GluR2-lacking receptors can mediate NMDAR-independent LTP [13], [16], [17], [18]. Consequently, a potential mechanism for NMDAR-independent learning and plasticity in the hippocampus is the expression and activation of GluR2-lacking (Ca^2+^-permeable) AMPARs.

The current study examined this possibility in mice with the GluR2 subunit of the AMPAR deleted in the CA1 region of the hippocampus and layer III of overlying cortex (GluR2-cKO mice). Electrophysiological analyses found that GluR2-cKO mice exhibit a novel form of LTP in CA1 that is mediated by Ca^2+^-permeable AMPARs and independent of NMDARs. Similar to previous studies, GluR2 deletion impaired memory on several hippocampus-dependent learning tasks. However, learning on NMDAR-independent versions of these tasks was normal in GluR2-cKO mice. Pharmacological studies revealed that NMDAR-independent learning required activation of Ca^2+^-permeable AMPARs in both knockout animals and wild-type mice. These data suggest that GluR2-lacking AMPARs play a unique role in NMDAR-independent learning.

## Results

### Tissue selective deletion of GluR2 in c-KO mice

**Figure 1:**
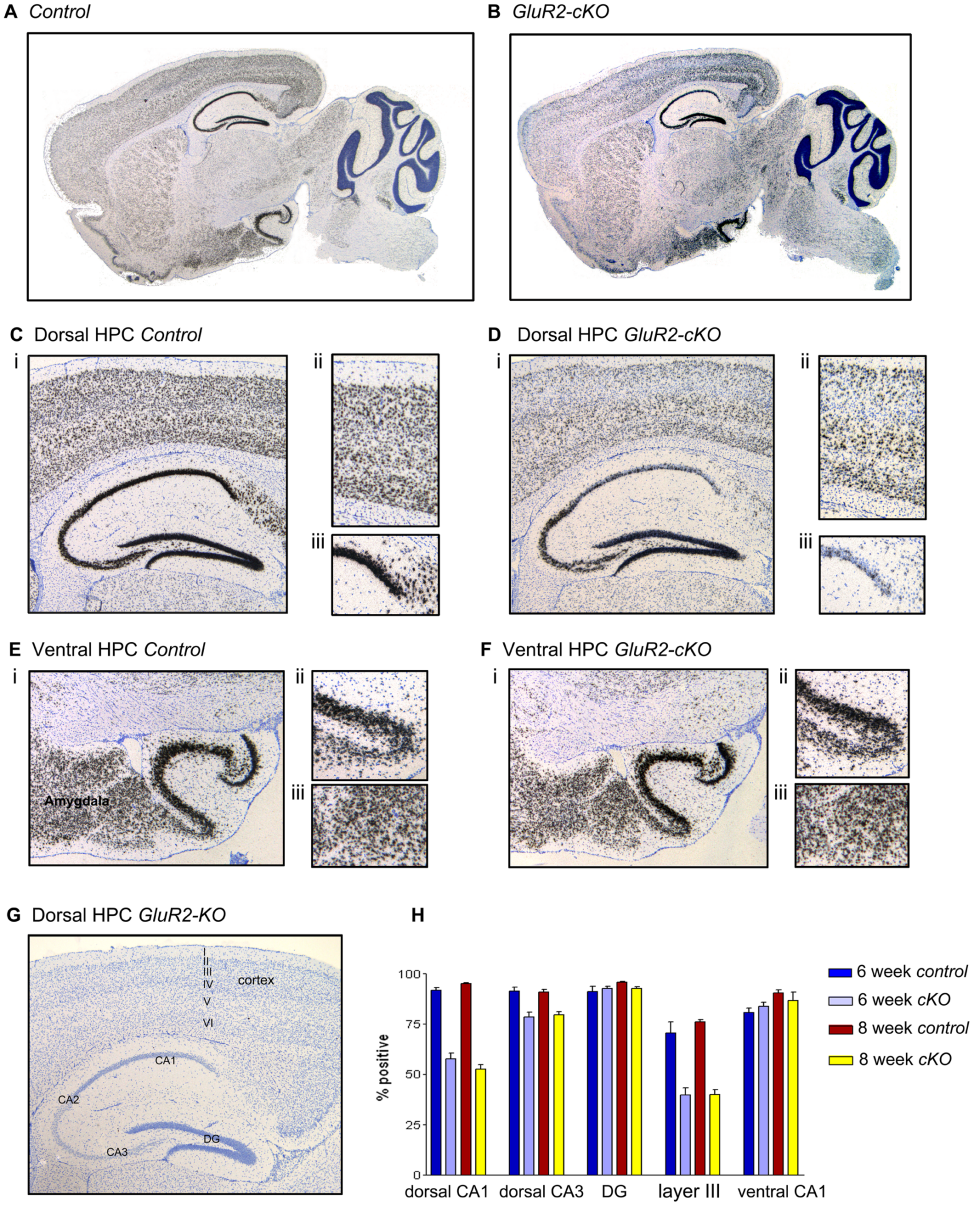
Conditional deletion of GluR2 in cKO mice. *In situ* hybridization with a GluR2 probe (black) demonstrates marked loss of GluR2 from the CA1 region of the dorsal hippocampus and from cortex layer III, but not from other brain regions, in the GluR2-cKO mice. *(* ***A–G*** *)* Representative images of GluR2 mRNA expression from brains of 8-week-old animals as evidenced by the pattern of *in situ* hybridization of a GluR2 probe. Shown are: *(* ***A*** *)* Whole brain of control animal; *(* ***B*** *)* Whole brain of GluR2-cKO animal; *(* ***C*** *)* Dorsal hippocampus and overlying cortex of control animal; *(* ***D*** *)* Dorsal hippocampus and overlying cortex GluR2-cKO animal; *(* ***E*** *)* Ventral hippocampus and amygdala of control animal; *(* ***F*** *)* Ventral hippocampus and amygdala of GluR2-cKO animal; *(* ***G*** *)* Dorsal hippocampus and overlying cortex of GluR2 global KO animal; *(* ***H*** *)* Percent GluR2 positive cells in brains taken from mice at 6 and 8 weeks age in dorsal hippocampus CA1 region, dorsal CA3 region, dorsal dentate gyrus (DG), cortex layer III, and in ventral hippocampus CA1 region. *(* ***C–G*** *)* (i) Low power images (magnification = 4×), *(* ***C–D*** *)* (ii) high power image of cortex (magnification = 10×), *(* ***C–D*** *)* (iii) and *(* ***E–F*** *)* (ii) high power image of hippocampus CA1 region (magnification = 10×), *(* ***E–F*** *)* (iii) high power image of amygdala adjacent to ventral hippocampus (magnification = 10×). All images couterstained with Nissl stain (blue). A description of the approach used to generate the GluR2-cKO and Glur2-KO mice as well the molecular characterization of these mice is shown in Fig. S1.

To study the effects of Ca^2+^-permeable AMPARs on plasticity and memory we generated a line of mice in which GluR2 was deleted from pyramidal cells of the CA1 region of the hippocampus as described in Text S1. In order to identify the loss of GluR2 mRNA we performed in situ hybridization on brain slices obtained from floxed mice (controls), GluR2-cKO mice and global GluR2-KO mice. Representative sagittal sections are shown in Fig. 1*A–B*. In the GluR2-cKO mice, there was significant loss of GluR2 in the dorsal hippocampus and in cortex layer III (Fig. 1*C–D*). When compared to control mice, the largest loss of GluR2 from the dorsal hippocampus of GluR2-cKO mice was seen in the CA1 region while minor loss of GluR2 was observed in CA3. In contrast to this conditional deletion, we observed a total loss of GluR2 mRNA from GluR2-KO mice, as expected (Fig. 1*G*). Expression of GluR2 in the ventral hippocampus (CA1, CA3 and dentate gyrus) was completely normal in GluR2-cKO mice (Fig. 1*E–F*). We also noted no difference in expression of GluR2 in the amygdala between GluR2-cKO mice and controls.

In order to quantify the loss of GluR2 mRNA, we analyzed the percentage of neurons that contain GluR2 mRNA at 6 and 8 weeks of age in control and GluR2-cKO mice in these brain regions (Fig. 1*H*). As expected, we observed a significant loss of GluR2 expression in dorsal CA1 pyramidal neurons in the GluR2-cKO mice at 6 weeks and 8 weeks of age compared to control mice of the same age (57.7±3.0% vs 91.8±1.4% at 6 weeks and 52.8±2.2% vs 95.2±0.4% at 8 weeks; p values<.05). Similarly, when cortex layer III neurons were counted, we observed a significant loss of GluR2 in the GluR2-cKO mice at 6 weeks and 8 weeks of age when compared to control mice of the same age (39.8±3.5% vs 70.6±5.5% at 6 weeks, and 40.0±2.2% vs 76.2±1.0% at 8 weeks; p values<.05). Meanwhile, in the dorsal CA3 region a modest but significant loss was seen at both time points when comparing GluR2-cKO mice to controls (78.5±2.6% vs 91.4±2.1% and 79.7±1.6% vs 91.0±1.3%; p values<.05). As expected, the dorsal dentate gyrus (DG) showed no loss in percentage expression (92.8±1.1% vs 91.3±2.6% and 92.7±0.9% vs 95.7±0.5%; p values>.05). We also found no statistically significant loss of GluR2 from pyramidal neurons in the CA1 region of the ventral hippocampus of the GluR2-cKO mice at 6 and 8 weeks of age when compared to controls of the same ages (83.9±1.9% vs 80.9±2.2% at 6 weeks and 86.7±4.1% vs 90.5±1.6% at 8 weeks; p values>.05).

In all subsequent experiments, we only used mice that were between 6 and 8 weeks of age. In order to assess if there was any change in the percentage of cells expressing GluR2 during this time period in mice within the same genotype, we compared percentage of cells that contained GluR2 mRNA at these two time points within each genotype, at all five anatomic regions analyzed. In the controls we observed no loss of GluR2 in dorsal hippocampus CA1 at 8 weeks compared to 6 weeks of age (p>.05). Importantly, it was also apparent that in the GluR2-cKO mice there was no further loss of GluR2 from dorsal CA1 pyramidal neurons between 6 and 8 weeks of age (p>.05). Likewise, the percentage of cortical layer III cells positive for GluR2 mRNA did not change between 6 and 8 weeks of age in the controls (p>.05), or, importantly, in the GluR2-cKO mice (p>.05). Nor was there any significant loss of cells positive for GluR2 in the GluR2-cKO mice between 6 weeks and 8 weeks of age in pyramidal neurons in the CA1 region of the ventral hippocampus, or in the DG, or CA3 regions of the dorsal hippocampus (p values >.05).

Based on these results we conclude that the majority of GluR2 loss is restricted to the dorsal hippocampus CA1 region and cortex layer III in the GluR2-cKO mice, with lesser loss in the dorsal CA3, and that expression levels are stable between 6 and 8 weeks of age in both genotypes in all five anatomical regions investigated.

### Altered GluR2 gene expression does not affect cell survival

**Figure 2:**
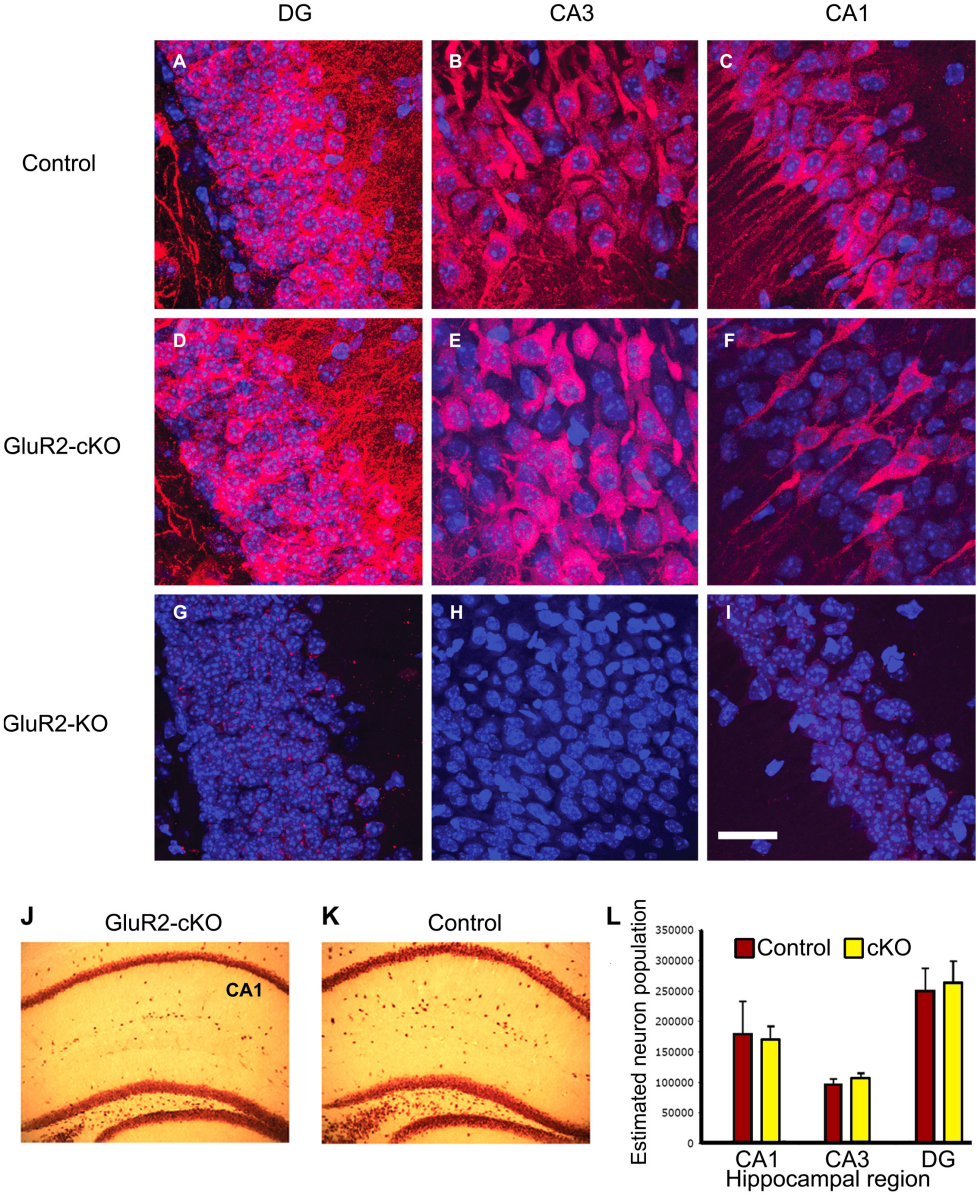
GluR2-cKO mice exhibit loss of GluR2 protein in CA1 and unchanged neuronal numbers. *(* ***A–I*** *)* Tissue from GluR2-cKO, control, and GluR2 global KO animals were immunohistochemically labeled for GluR2 (red) and counterstained with DAPI (blue). Confocal micrographs were obtained of the DG, CA3 and CA1 regions in the hippocampus. Scale = 50 µm. *(* ***J–K*** *)* Tissue from GluR2-cKO and control animals were immunohistochemically labeled for the neuronal marker NeuN for stereological estimation of the neuronal population. *(* ***L*** *)* Quantification of neuronal populations in the DG, CA3 and CA1 regions using the Stereo Investigator. Values as mean ± s.e.m. Note that the knockout of GluR2 did not lead to changes in the expression of other glutamate receptor subunits (See Fig. S2 and Table S1).

We next confirmed that loss of hippocampal GluR2 mRNA results in loss of GluR2 protein in GluR2-cKO mice. Immunohistochemical staining (Fig. 2) with an anti-GluR2 antibody revealed that, when compared with the control mice (Fig. 2*A–C*), the GluR2-cKO mice showed profound loss of GluR2 protein from cells in the CA1 region (Fig. 2*F*), little loss of GluR2 from the CA3 region (Fig. 2*E*) and no evident loss of GluR2 protein from the DG (Fig. 2*D*). Meanwhile GluR2 was completely absent from the hippocampus of GluR2 global KO mice (Fig. 2*G–I*).

It is possible that the loss of GluR2 protein could lead to increased calcium influx into cells through AMPARs which might lead to cell death. To assess whether there was loss of hippocampal neurons in 8 week old mice, we stained sections using an antibody to NeuN, which is a marker of mature neurons (Fig. 2*J, K*), and we employed stereology [19] to count the number of neurons in dorsal hippocampus between bregma AP -1.34 mm and bregma AP -2.06 mm in 9 control and 6 GluR2-cKO mice. There was no difference in the number of neurons in GluR2-cKO mice compared with controls (Fig. 2*L*). These combined results demonstrate that while GluR2 expression is significantly reduced from the expected regions of the dorsal hippocampus of mutant mice this loss does not lead to cell death in mice at eight weeks of age. In addition, knockout of GluR2 did not affect the expression of other glutamate receptor subunits (Fig. S2; for primer sequences see Table S1).

### Loss of GluR2 leads to enhanced LTP at synapses

A number of studies have characterized the effects of GluR2 deletion on synaptic transmission in the hippocampus [16], [17]. Therefore, we first confirmed that our GluR2-cKO mice showed similar changes (see Text S1). As predicted from previous work, synaptic transmission was sensitive to blockers of GluR2-lacking AMPARs (Fig. S3), input/output functions were right-shifted (Fig. S3 *B*) and there was little change in paired-pulse facilitation, (Fig. S3 *C*).

**Figure 3:**
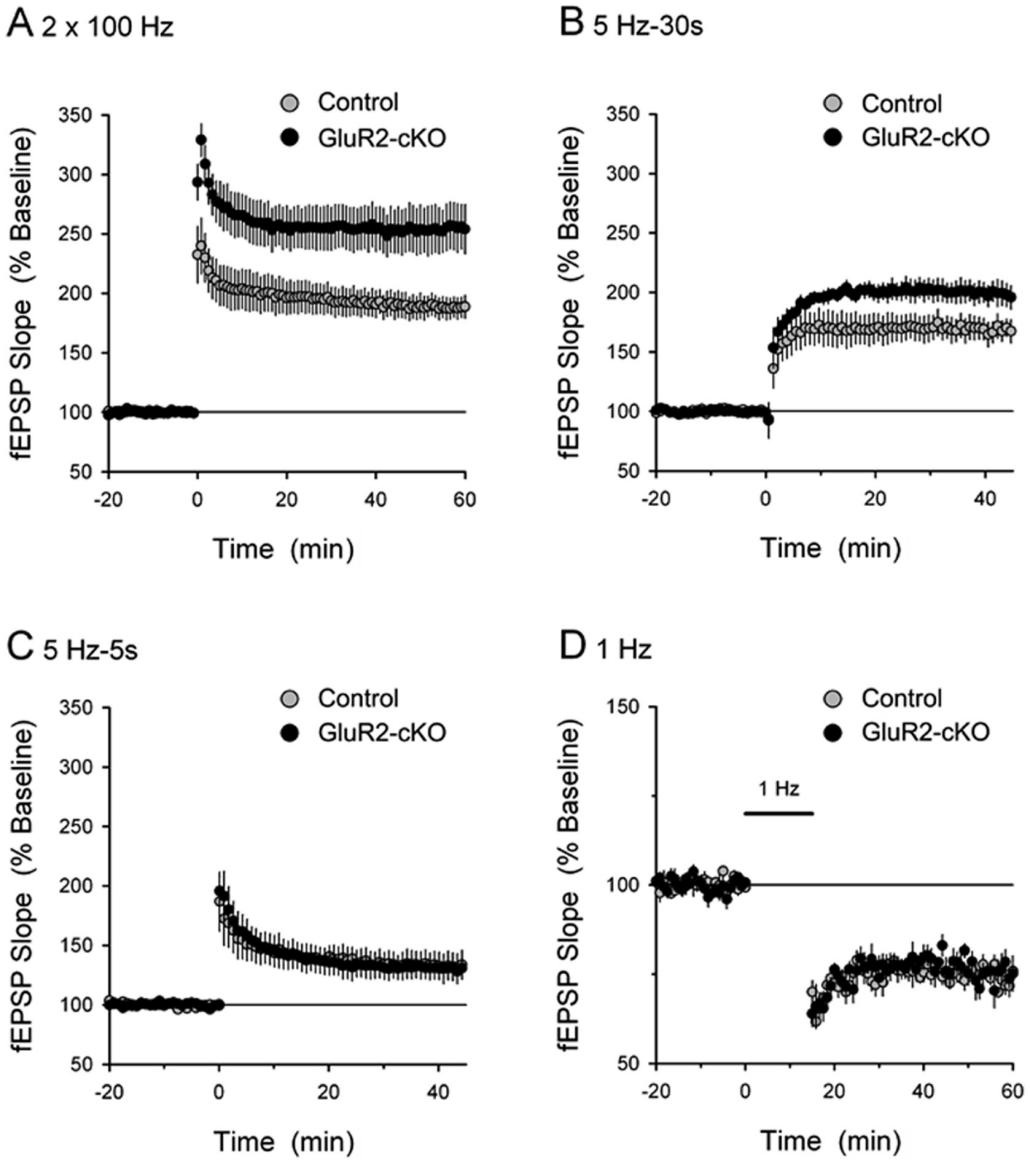
LTP is enhanced in GluR2-cKO mice. (***A***) HFS stimulation-induced LTP is enhanced in slices from GluR2-cKO mice. LTP was induced at time = 0 with two, 1 second long trains of 100 Hz stimulation (inter-train interval = 10 sec). (***B***) A 30 second long train of 5 Hz stimulation induces significantly larger LTP in slices from GluR2-cKO mice. 45 minutes post-5 Hz stimulation (delivered at time = 0) fEPSPs were potentiated to 168±9% of baseline in control slices (gray symbols, n = 5) and were potentiated to 198±9% of baseline in slices from GluR2-cKOs (black symbols, n = 5). (***C***) A short train of 5 Hz stimulation (5 sec) induces similar levels of LTP in control (gray symbols, n = 5) and GluR2-cKO slices (black symbols, n = 5). (***D***) LTD is normal in GluR2-cKO slices. LTD was induced using a 15 minute long train of 1 Hz stimulation (indicated by the bar). The magnitude of LTD seen 60 minutes after the start of 1 Hz stimulation is similar in wild type (gray symbols, n = 6) and GluR2-cKO slices (black symbols, n = 5). Baseline synaptic transmission was altered as predicted in the GluR2-cKO mice (see Fig. S3).

The induction of LTP by a conventional high-frequency stimulation (HFS) protocol was significantly enhanced in the CA1 region of GluR2-cKO mice (60 minutes post-HFS, fEPSPs were potentiated to 255±19% of baseline in GluR2-cKO mice, n = 7, compared to 188±9% of baseline in control littermates, n = 8, p<.05) (Fig. 3*A*). Consistent with the notion that the larger LTP observed in GluR2-cKOs is associated with Ca^2+^ influx via GluR2-lacking AMPARs, HFS induced a significant LTP in slices from GluR2-cKO mice bathed in ACSF containing the NMDAR antagonist D-APV (100 µM; 60 minutes post-HFS fEPSPs were potentiated to 134±4% of baseline in cKO slices, n = 4, compared to 110±3% of baseline in control slices, n = 4, p<05). Nonetheless, the amount of potentiation was still reduced by D-APV in both groups (p<.05) suggesting that NMDARs contribute to LTP in GluR2-cKO mice and controls. While the induction of LTP by a less robust pattern of synaptic stimulation (30 seconds of 5 Hz stimulation) was also significantly enhanced in GluR2-cKO mice (Fig. 3*B*), a shorter train of 5 Hz stimulation, near to the threshold for LTP induction in control mice, produced similar levels of LTP in slices from GluR2-cKOs and controls (Fig. 3*C*). When LTD was induced by a long train of low-frequency stimulation (1 Hz for 15 min) similar levels of decreased responding were observed in GluR2-cKO and control slices (Fig. 3*D*). Together, these findings are consistent with previous studies showing that the absence of GluR2 AMPAR subunits enhances the induction of LTP by some patterns of synaptic stimulation with little or no effect on LTD [16], [17], however see [20]. It is notable that other studies also provide data that is consistent with our findings, in that they demonstrate that Ca^2+^-permeable AMPARs alter NMDAR dependency of HFS-induced CA1 LTP [21], [22], [23].

### GluR2-cKO mice express a unique form of synaptic plasticity

Hebbian plasticity refers to strengthening that is contingent upon the concurrent release of neurotransmitter and post-synaptic membrane depolarization (see Text S1). The current-voltage (I/V) relations for NMDARs are such that channel conductance is low at negative membrane potentials and increases with membrane depolarization as the voltage-dependent Mg^2+^ ion block of the channel is relieved. Consequently, LTP is induced by NMDARs when the post-synaptic membrane is depolarized, coincident with activation of the receptor by glutamate. Hence LTP that is mediated by the NMDAR is referred to as Hebbian.

By contrast, the current-voltage (I/V) relations for GluR2-lacking (Ca^2+^-permeable) AMPAR channels is inwardly rectifying, so that channel conductance and Ca^2+^ influx through GluR2-lacking AMPARs is most profound at negative (i.e. hyperpolarized) membrane potentials. Consequently, LTP mediated by GluR2-lacking (Ca^2+^-permeable) AMPARs can be generated when presynaptic fiber stimulation is paired with hyperpolarization of the postsynaptic membrane [23].

**Figure 4:**
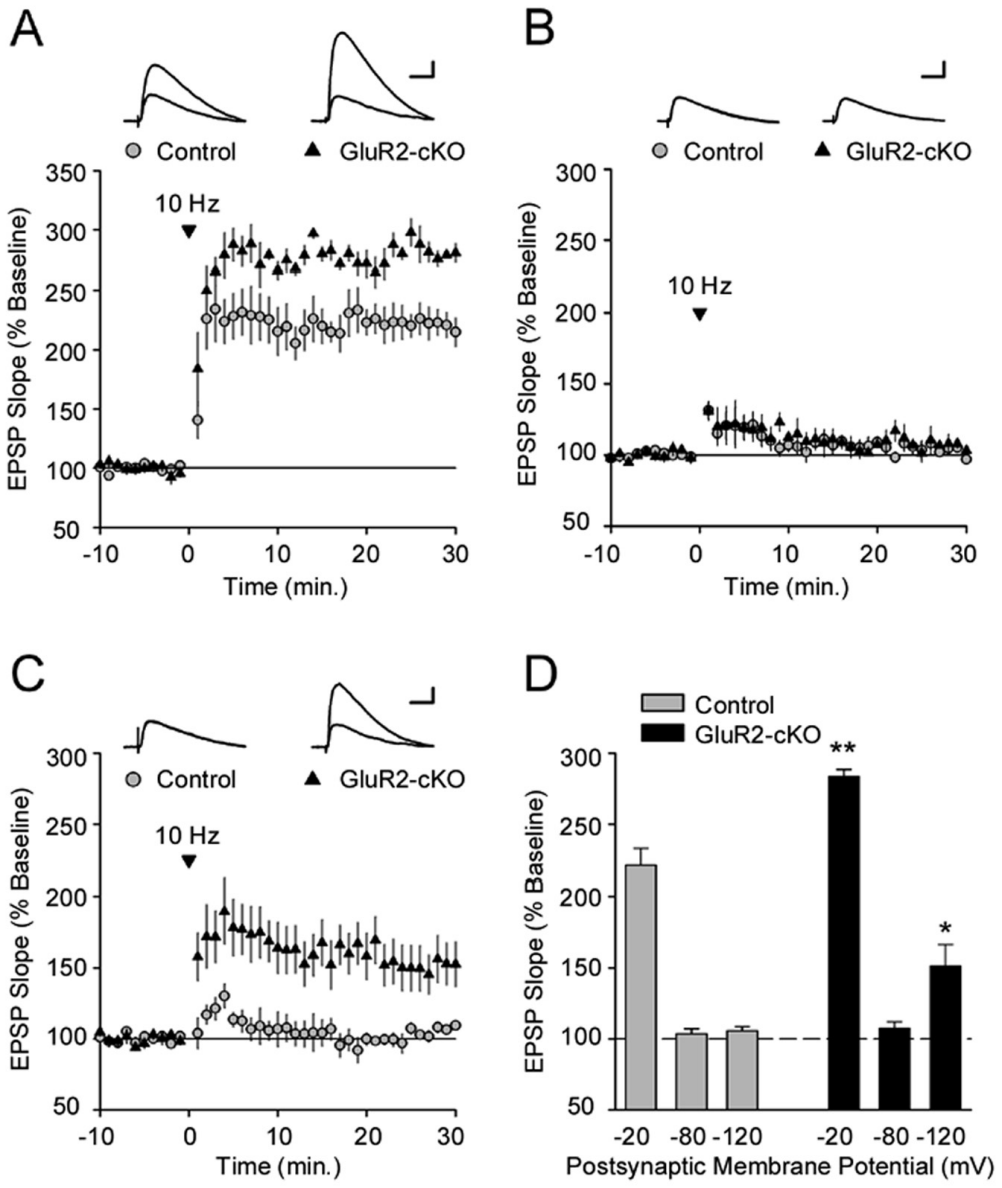
LTP can be induced in GluR2-cKO mice at both hyperpolarized and depolarized membrane potentials. (***A***) A Hebbian LTP induction protocol (10 Hz presynaptic fiber stimulation paired with postsynaptic depolarization) induces LTP in cells from both control (gray circles, n = 7 cells) and GluR2-cKO slices (black triangles, n = 7 cells). (***B***) 10 Hz stimulation at resting membrane potential failed to induce LTP in pyramidal cells from both wild type (n = 14 cells from 4 mice) and GluR2-cKO mice (n = 11 cells from 3 mice). (***C***) A 10 Hz stimulation paired with postsynaptic hyperpolarization has no effect on synaptic transmission in control cells (open symbols, n = 12 cells) but induces LTP in cells from GluR2-cKO mice (black symbols, n = 11 cells). The traces shown in *A*, *B*, and *C* are superimposed EPSPs recorded during baseline and 30 minutes post-pairing in a control and GluR2-cKO cell. Scale bars are 20 milliseconds and 5 mV. (***D***) Histograms show the amount of potentiation present 30 minutes post-pairing in control (gray bars) and GluR2-cKO cells (black bars).

In the next experiment we examined whether the loss of GluR2 in CA1 pyramidal neurons enables the expression of LTP at synapses onto these cells when they are hyperpolarized. In these experiments whole-cell current-clamp recordings were used to pair short trains of presynaptic fiber stimulation (100 pulses at 10 Hz) with either depolarization or hyperpolarization of the postsynaptic cells to approximately −20 or −120 mV, respectively. As shown in Fig. 4*A*, a “Hebbian” protocol (i.e. pairing a train of 10 Hz synaptic stimulation with postsynaptic depolarization) induced robust LTP in control cells (30 minutes post-pairing EPSPs were potentiated to 221±12% of baseline, n = 5). In pyramidal cells from GluR2-cKO mice this same pairing protocol also induced robust LTP that was significantly larger than that seen in cells from control slices (EPSPs were potentiated to 283±5% of baseline, n = 4, p<.05 compared to controls). In contrast, pairing 10 Hz stimulation with postsynaptic hyperpolarization to approximately −120 mV (Fig. 4*C*) never induced significant LTP in cells from control mice (0 out of 12 cells, 30 minutes post-pairing EPSPs were 106±3% of baseline, n = 6), however in approximately half of the cells (5 out of 11 cells) we recorded from in slices from GluR2-cKO mice this pairing protocol did induce significant LTP (across all cells EPSPs were potentiated to 150±15% of baseline, n = 9, p<0.05 compared to control). Stimulation in the absence of postsynaptic hyperpolarization did not produce potentiation in GluR2-cKO mice (Fig. 4*B*). This demonstrates that LTP in these animals can be induced via a second, separate mechanism from classical Hebbian LTP induction.

In summary, the experiments above demonstrate that LTP in the CA1 region of our mutants is dependent on both NMDARs and Ca^2+^-permeable GluR2-lacking AMPARs. The next experiments examined the functional effects of adding AMPAR-dependent plasticity to CA1 pyramidal cells.

### Contextual fear conditioning is impaired in GluR2-cKO mice

**Figure 5:**
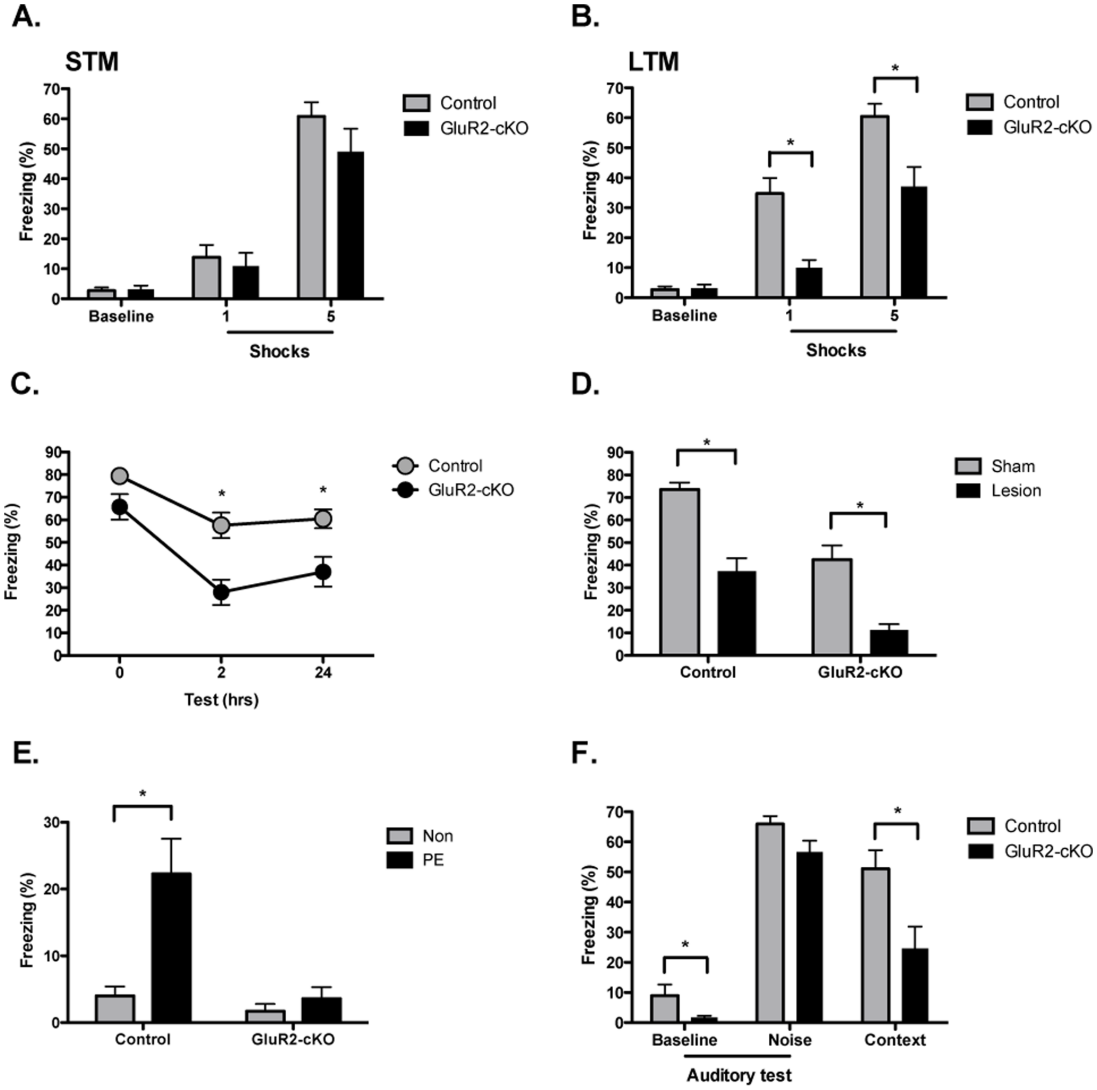
Deletion of GluR2 in CA1 impairs context fear. (***A***) Controls and GluR2-cKO mice showed similar increases in context fear immediately after 1 (controls n = 13, GluR2-cKO n = 13) or 5 (controls n = 21, GluR2-cKO n = 12) shocks. (***B***) Controls showed more context fear than GluR2-cKO mice 24-hours after training. This difference was observed in both the 1 and 5 shock groups. Baseline freezing levels from the training session (prior to shock delivery) are shown for comparison. (***C***) To examine the time course of memory loss we tested separate groups of animals at three different time points after training. Memory was not reduced in GluR2-cKO mice immediately after training (controls n = 10, GluR2-cKO n = 12) but was impaired at 2 (controls n = 9, GluR2-cKO n = 14) and 24 (controls n = 21, GluR2-cKO n = 12) hours. (***D***) Excitotoxic hippocampus lesions made 1 day after training produced amnesia for context fear in both controls (sham n = 21, lesion n = 8) and GluR2-cKO mice (sham n = 15, lesion n = 13) (***E***) The ability to form a long-term memory of the context in the absence of shock was examined using a context pre-exposure (PE) procedure. PE produced robust context learning in control mice (n = 11) relative to non-exposed animals (n = 15). In contrast, pre-exposed GluR2-cKO mice (n = 12) showed less freezing than control mice and were not different from cKO mice not exposed to the context (n = 12) (***F***) Auditory fear conditioning produced equivalent freezing increases in controls (n = 15) and cKOs (n = 18) during the white noise relative to baseline. GluR2-cKO mice were impaired during the context test conducted the next day. Error bars represent ± SEM and * indicates statistical significance (p<.05).

We first examined contextual fear conditioning, a form of learning known to depend on the hippocampus and NMDAR-mediated plasticity in CA1 [3], [7], [24], [25]. Mice were placed in a novel environment and allowed to explore for two minutes before receiving either 1 or 5 footshocks. Control mice and GluR2-cKOs showed significant and equivalent increases in freezing immediately after shock relative to the baseline period (no effect of genotype F (1,55)  = 1.48, p>.05, effect of shock number F (1, 55)  = 67.93, p<.05, no genotype x shock number interaction F<1, effect of period (baseline vs. shock) F (1, 55)  = 113.09, p<0.05), no period x genotype interaction F (1, 55)  = 2.125, p>.05, period x shock number interaction F (1, 55)  = 49.85, p<.05, no period x genotype x shock number interaction F<1) (Fig. 5*A*). There are substantial data indicating that freezing during this period is entirely a conditional response to contextual stimuli that have become associated with shock. Therefore, similar levels of post-shock freezing suggest that short-term memory is intact in GluR2-cKO animals [26], [27] (see also Text S1). The same mice were brought back to the context 24 hours later to test for long-term memory (Fig. 5*B*). During this test, GluR2-cKOs froze significantly less than control mice (main effect of genotype F (1, 55)  = 24.69, p<0.05) both in the 1 and 5 shock groups (no genotype x shock number interaction F<1). Baseline freezing levels from the training session (prior to shock delivery) are shown for comparison. These data demonstrate that deletion of GluR2 in the CA1 region impairs long-term memory for context fear.

In the next experiment, we examined the time course of memory loss by testing animals immediately, 2 hours or 24 hours after training (Fig. 5*C*). Based on the results above, we predicted that memory in GluR2-cKO mice would be normal immediately after training but impaired at longer delays. To test this idea we conducted a set of planned contrasts (Fisher's PLSD) between controls and GluR2-cKOs that revealed memory was intact immediately after training (p>.05) but significantly impaired 2 hours and 24 hours later (p values <0.05). The fact that we observed normal post-shock freezing in our first two experiments and in our subsequent c-fos experiment strongly suggests that short-term memory is intact in GluR2-cKO mice. These data are in line with reports that place field stability is significantly impaired in global GluR2 knockout mice [28].

To ensure that controls and GluR2-cKO mice were using the dorsal hippocampus to store context fear memories we selectively removed this structure 1 day after training (histology in Fig. S4 *D*). Previous studies have shown that post-training lesions of the dorsal hippocampus severely impair memory for context fear in both rats and mice [24], [29], [30], [31]. Consistent with these results, we found that controls and GluR2-cKOs with dorsal hippocampus lesions showed significantly less context fear than sham-operated animals (main effect of lesion, (F (1,53)  = 49.49, p<.05), main effect of genotype, (F (1, 53)  = 35.63. p<.05), no genotype x lesion interaction, F<1) (Fig. 5*D*). This demonstrates that the dorsal hippocampus is used to store and retrieve context fear memory in our experiments.

We next determined if GluR2-cKO mice were able form a long-term context memory in the absence of shock. To do this we used a context pre-exposure procedure that has previously been shown to depend on the hippocampus and NMDAR activation [32], [33]. In this procedure, animals learn about the context prior to fear conditioning and have to retain this information across a 24-hour period [29], [34], [35]. On day 1, half of the mice were pre-exposed to the training context for 10 minutes (in the absence of shock) while the other animals remained in their homecages. The next day, all animals were trained with an immediate shock delivered 5 seconds after placement in the context. Without pre-exposure, this short interval does not provide enough time to learn about the context before shock is delivered. Consistent with this fact, pre-exposed control animals showed considerably more context fear than non-exposed mice (Fig. 5*E*). In contrast, pre-exposed GluR2-cKOs did not benefit from this experience (main effect of genotype (F (1, 46)  = 16.07, p<.05, main effect of exposure (F (1, 46)  = 14.77, p<.05), significant genotype x exposure interaction (F (1, 46)  = 9.874, p<.05). Post-hoc tests revealed that pre-exposed control mice froze significantly more than pre-exposed GluR2-cKO animals (p<.05). These results demonstrate that GluR2-cKO mice have an impaired ability to form long-term memories of the context.

To determine if the GluR2-cKO deficit in fear conditioning was specific to context fear we also examined hippocampus-independent auditory fear conditioning [24], [30], [36]. This type of conditioning depends on NMDAR activation in the amygdala [37]. Mice were trained with 5 white noise-shock pairings and then received an auditory test in a novel environment 24 hours later (Fig. 5*F*). During the baseline period of this test, GluR2-cKO mice exhibited less generalized fear to the novel environment (main effect of genotype F (1, 31)  = 4.6, p<0.05), a phenotype consistent with their overall reduction in context fear. Despite this fact, GluR2-cKO mice showed robust increases in freezing during white noise presentations that did not differ from controls (effect of period (baseline vs. tone) F (1, 31)  = 434.93, p<0.05, main effect of genotype (F (1, 31)  = 7.18, p<.05, no period x genotype interaction F<1). Post-hoc tests revealed that white noise freezing levels were similar in controls and GluR2-cKO mice (p>.05). The same knockout animals exhibited significantly less context fear than controls (main effect of genotype F (1, 31)  = 9.89, p<0.05) when tested in the training environment the next day (Fig. 5*F*). These results demonstrate that the loss of GluR2 in CA1 selectively impairs context fear (see Text S1 for a more detailed discussion).

Lastly, we assessed motor function using rotarod and openfield tests (Fig. S4 *A* and S4 *B*) and pain processing by analyzing shock reactivity (Fig. S4 *C*) and found that them to be normal in GluR2-cKO mice. This indicates that the reduced context fear observed in these animals does not result from hyperactivity or reduced pain sensation. These data are also consistent with the fact that GluR2-cKO mice show normal short-term memory for context fear (Fig. 5*A and 5C*). Intact short-term memory suggests that context exploration and pain processing are normal [27], [38].

### Spatial learning and memory are impaired in GluR2-cKO mice

**Figure 6:**
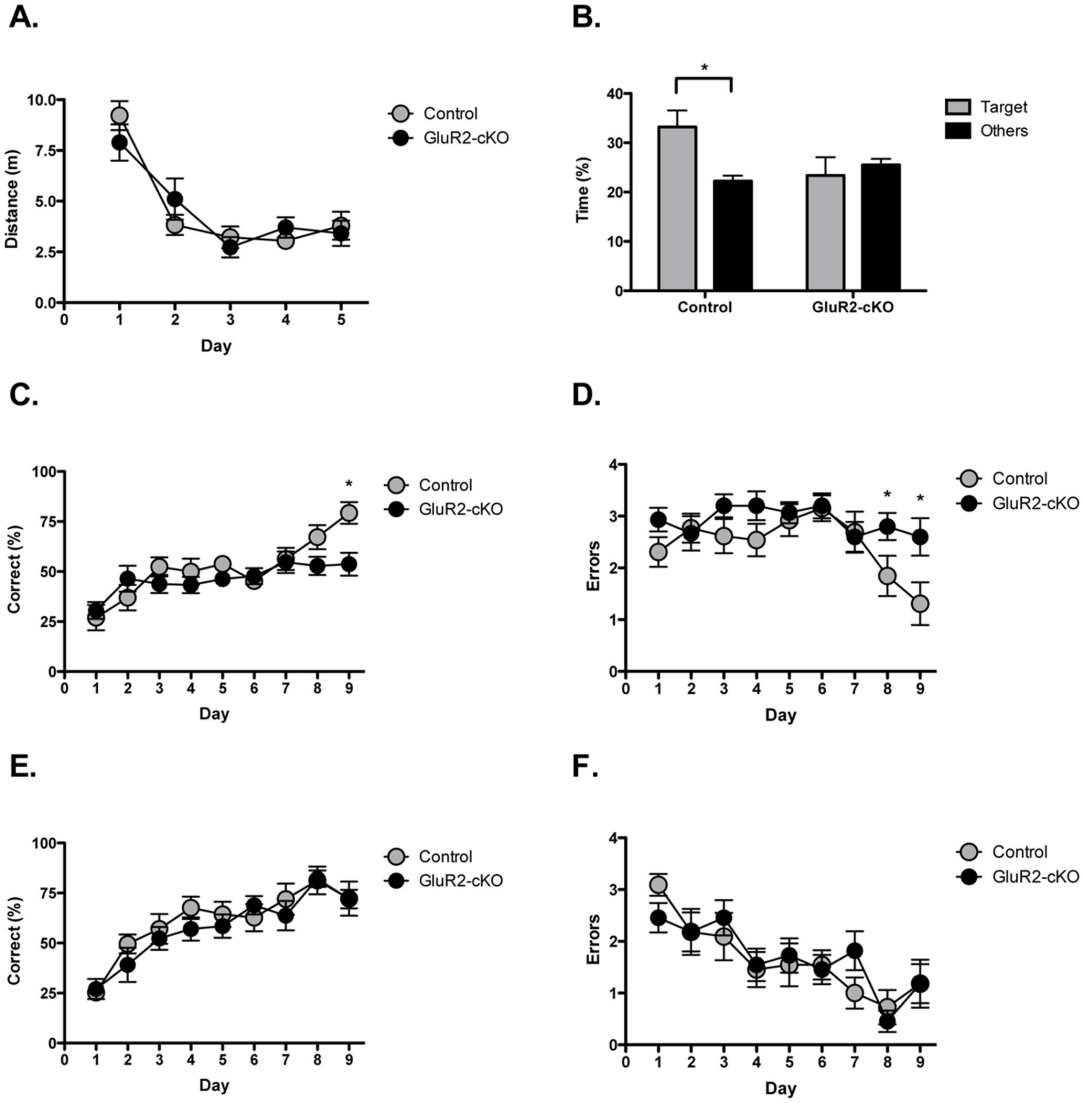
Long-term spatial memory is impaired in GluR2-cKO mice. *(* ***A*** *)* Controls (n = 16) and GluR2-cKO (n = 12) mice traveled the same distance in the watermaze to the find the platform across training days. *(* ***B*** *)* Control mice spent more time in the target quadrant then the other quadrants during the watermaze probe test. In contrast, GluR2-cKO mice spent an equivalent amount of time in all quadrants during the probe test. *(* ***C*** *)* Controls (n = 13) made a higher percentage of correct responses than GluR2-cKO mice (n = 15) on the reference memory version of the radial arm maze. *(* ***D*** *)* Controls showed a reduction in the number of errors (re-entries) across days on the reference memory version of the radial arm maze while GluR2-cKO mice did not *(* ***E*** *)* The percentage of correct responses on the working memory version (win-shift) of the radial arm maze was the same in controls (n = 11) and GluR2-cKO mice (n = 11) *(* ***F*** *)* Controls and GluR2-cKO mice showed equivalent reductions in the number of errors made across days in the working memory version of the radial arm maze. Error bars represent ± SEM and * indicates statistical significance (p<.05).

Spatial learning in the Morris watermaze is dependent on NMDAR activation in the hippocampus [2], [4]. We trained GluR2-cKO mice on a fixed-visible version of this task that produces robust spatial memory and facilitates procedural learning [39]. Across training days, both controls and GluR2-cKO mice showed a reduction in the distance traveled to reach the platform (main effect of day F (4, 104)  = 22.299, p<0.05, no effect of genotype (F<1), no day x genotype interaction (F (4, 104)  = 1.119, p>.05) (Fig. 6*A*). Spatial memory was assessed on day 6 by administering a 60 s probe test with the platform removed. Control mice showed selective searching in the target quadrant where the platform was located during training compared to the other quadrants (main effect of quadrant, F (1, 15)  = 6.102, p<.05) (Fig. 6*B*). In contrast, GluR2-cKO mice searched equally in all quadrants suggesting they did not learn the spatial location of the platform (no effect of quadrant, F<1). These data suggest that deletion of GluR2 in the CA1 region of the hippocampus impairs the formation of spatial memory.

Long-term spatial memory was also assessed using the reference memory version of the radial maze [40], [41], [42], [43]. In this task, mice were required to remember the spatial location of 4 baited arms across a 24-hour period. The same arms were baited during each session and the mice were trained for 9 consecutive days. There was an increase in the percentage of correct choices across training days (main effect of day F (8, 208)  = 9.94, p<0.05) that was larger in control animals than GluR2-cKOs (significant day x genotype interaction F (8, 208)  = 2.31, p<0.05, no effect of genotype F (1, 26)  = 2.849, p>.05) (Fig. 6*C*). Post-hoc tests (Fisher's PLSD) revealed that control mice performed significantly better than knockout animals on the last day of training (p<0.05). Control mice also showed a significant decrease in errors (i.e. visits to unbaited arms) across days (main effect of day F (8, 104)  = 2.52, p<0.05) while GluR2-cKO mice did not (no effect of day F<1) (Fig. 6*D*). Post-hoc tests (Fisher's PLSD) showed that control mice made significantly fewer errors on days 8 and 9 than GluR2-cKO animals (p<0.05). These results are consistent with our watermaze data and demonstrate that the deletion of GluR2 in CA1 impairs the formation of long-term spatial memories.

In the next experiment we examined spatial working memory using the win-shift version of the radial maze [40], [41], [44], [45]. On this version of the task, animals were required to remember the spatial location of 4 baited arms across a 2-minute period. New arms were randomly chosen on each day to eliminate the contribution of long-term spatial memory. We found that controls and GluR2-cKO mice showed significant (main effect of day F (8, 160)  = 15.36, p<0.05) and equivalent (no effect of genotype, F<1, no day x genotype interaction F<1) increases in the percentage of correct choices across training days (Fig. 6*E*). In addition, the number of errors (i.e. visits to unbaited arms) made by controls and cKO mice decreased across days (main effect of day F (8, 160)  = 7.65, p<0.05) and were not different between groups (no day x genotype interaction F<1) (Fig. 6*F*). To demonstrate that the mice were not forming long-term spatial memories we increased the interval between phases (2–480 min) and found a systematic decrease in the performance of controls and cKO animals (main effect of time F (5, 100)  = 15.55, p<0.05) that did not differ between groups (no time x genotype interaction F<1) (data not shown). Animals returned to naive levels of performance at the longest interval and made the same number of errors as they did on the first day of training (no effect of session (first vs. last) F (1, 20)  = 1.08, p>0.05). These results suggest that selective deletion of GluR2 in CA1 does not impair spatial working memory. This finding is consistent with recent data showing that NMDAR-dependent plasticity in CA1 is not required for the retention of spatial information across short intervals [46].

Thus, in addition to showing deficits in contextual fear conditioning GluR2-cKO animals exhibit impaired long-term memory in spatial learning tasks that are known to require NMDAR activation in the CA1 region of the hippocampus. Therefore, our study is the first to demonstrate that hippocampus-dependent learning impairments in GluR2 knockout mice can be produced by targeted deletion of this subunit in the CA1 region. This deficit does not result from impaired NMDAR-dependent LTP as our electrophysiology experiments demonstrate that D-APV significantly reduces potentiation in GluR2-cKO mice.

### Immediate early gene expression is normal in GluR2-cKO mice

**Figure 7:**
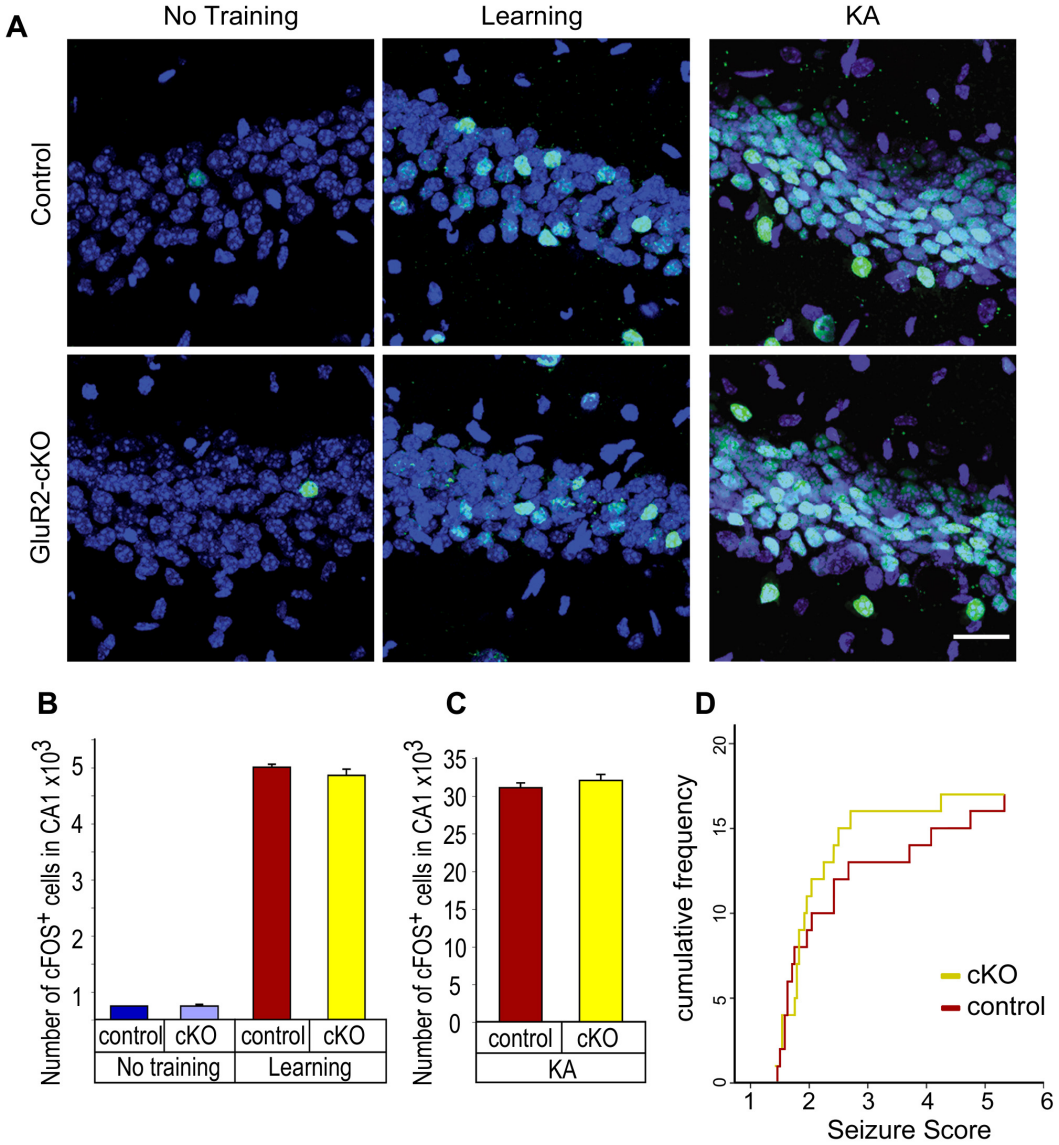
Knockout of GluR2 in CA1 does not affect immediate early gene expression or seizure susceptibility. (**A**) Confocal micrographs of cFOS-positive cells (green), detected by immunohistochemical staining, counterstained with nuclear marker DAPI (blue) in the CA1 of control and GluR2-cKO mice prior to training (‘no training’), following training (‘learning)’, and following kainate-induced seizures (KA) (n = 3 animals per group per genotype). Scale bar = 30 mm. (**B**) Quantitative analysis of cFOS-positive cells in the CA1 using stereology in control and GluR2-cKO mice that had no training or following training. Values shown as mean ± s.e.m. (**C**) Quantitative analysis of cFOS-positive cells in the CA1 using stereology in control and GluR2-cKO mice that had received a single i.p. injection of 25 mg/kg KA. Values shown as mean ± s.e.m. (**D**) Comparison of seizure susceptibility in control and GluR2-cKO animals. Seizure score is defined as the total seizure score divided by the number of observations and plotted as a cumulative frequency step graph – see experimental methods regarding seizure score scale. As the data were not normally distributed they were analyzed by Mann-Whitney. The Benjamini-Hochberg FDR correction was applied to correct for the multiple t-tests performed. No significant difference between curves was observed.

It is possible that the learning impairments observed in our GluR2-cKO mice result from reduced synaptic transmission and/or excitability and not altered plasticity. We addressed this issue by determining if CA1 neurons are similarly engaged by fear conditioning in controls and GluR2-cKO mice. To do this we examined the expression of c-fos, an immediate early gene that is an indicator of neural activity and whose expression is significantly increased in the CA1 region of the hippocampus following context fear conditioning [39], [47], [48], [49], [50]. Homecage controls were compared to mice that received 5 unsignaled shocks (identical to the training procedures above). Conditioned mice showed robust short-term memory at the end of training that did not differ between controls and GluR2-cKO animals (no effect of genotype F<1). Ninety minutes later we sacrificed the animals, froze their brains on dry ice and performed immunohistochemical staining to determine the level of c-fos expression in the CA1 region of the hippocampus (Fig. 7*A*). Controls and GluR2-cKO mice that remained in their homecages showed similar levels of expression (F<1). Following fear conditioning, there was a robust increase in c-fos expression that also did not differ between control and GluR2-cKOs (Fig. 7*B*) (main effect of training F (1, 8)  = 4439.43, p<0.05, no training x genotype interaction F (1, 8)  = 1.17, p>0.05). These results demonstrate that context fear conditioning produces normal activation of CA1 neurons in GluR2-cKO animals. We also looked at maximal activation following kainate-induced seizures (Fig. 7*C*). Once again there was no difference in the level of c-fos expression between controls and GluR2-cKO animals (no effect of genotype F<1). In addition, no difference in seizure susceptibility was observed (Fig. 7*D*). Together, these results suggest that the learning deficits observed in our GluR2-cKO mice are not due to reduced neural activity in the CA1 region of the hippocampus.

### NMDAR-independent learning is not impaired in GluR2-cKO mice

Our results demonstrate that deletion of GluR2 in the CA1 region of the hippocampus produces AMPAR-mediated plasticity that impairs learning and memory. However, AMPAR-mediated plasticity may not be detrimental to all types of learning. In fact, a number of different experiences (e.g. learning, drug exposure, sensory deprivation) increase the expression of GluR2-lacking Ca^2+^-permeable AMPARs [9], [51], [52], [53], [54]. It is possible, therefore, that the experience-dependent expression of these receptors plays a functional role in subsequent plasticity and learning (i.e. experience-dependent learning). The following experiments examined this idea.

**Figure 8:**
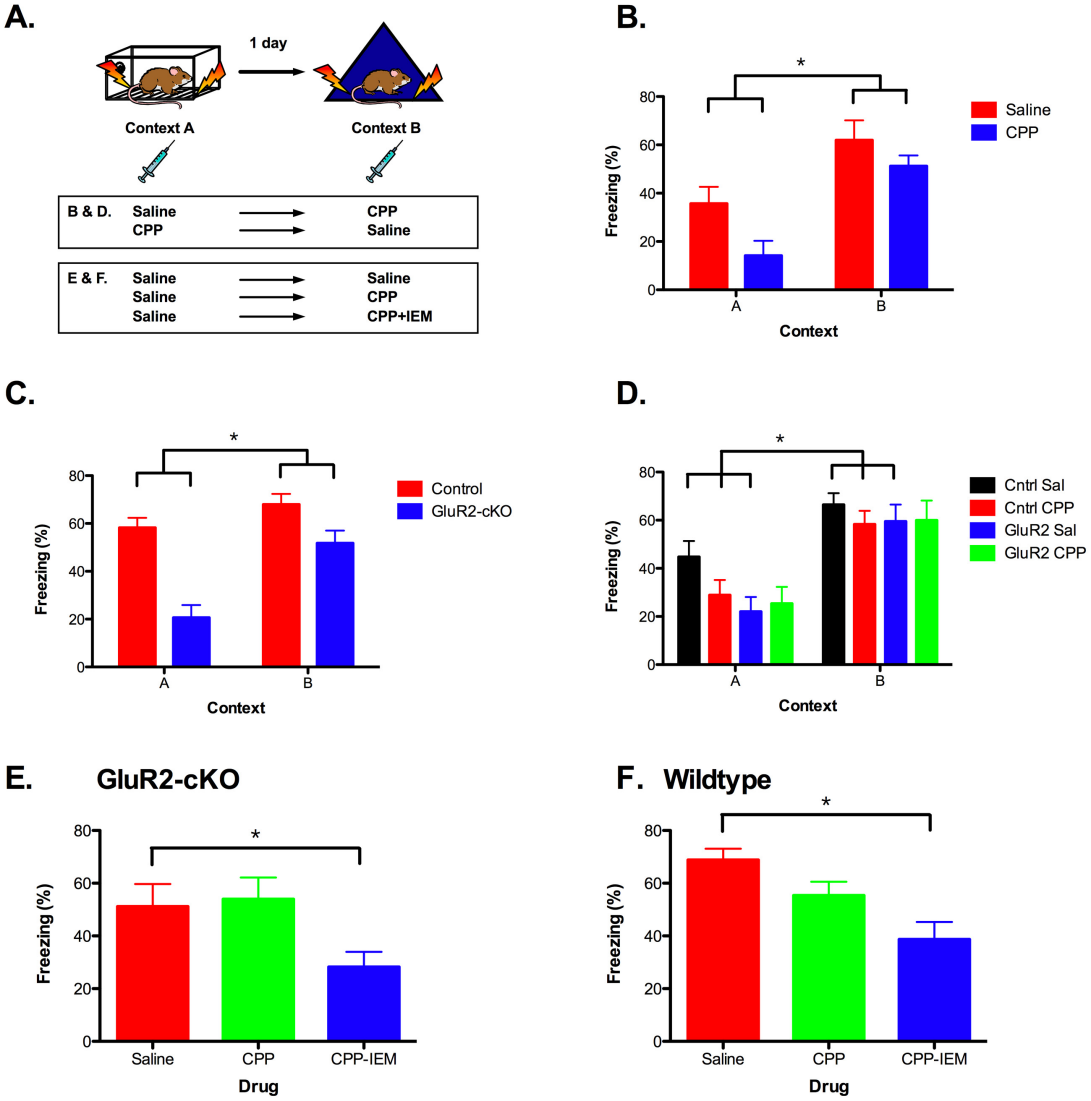
NMDAR and GluR2-lacking AMPAR make distinct contributions to learning. (***A***) Experimental design. Animals were trained in context A on day 1 and context B on day 2. Mice were then tested for freezing in context B and context A on days 3 and 4 (***B***) The NMDAR antagonist CPP was given prior to training in context A in one group of mice and prior to context B in a second group of mice. CPP was more effective at blocking learning in context A than context B (saline n = 8, CPP n = 8). (***C***) Deletion of GluR2 was more effective at blocking learning in context A than context B (control n = 26, GluR2-cKO n = 23) (***D***) Learning in context A was impaired in GluR2-cKO mice and animals receiving CPP (controls saline n = 16, GluR2-cKO saline n = 7, controls CPP n = 21, GluR2-cKO CPP n = 13) while learning in context B was unaffected by either of these manipulations. (***E***) GluR2-cKO were trained in context A and then received injections of saline, CPP or CPP +IEM-1460 (a Ca^2+^-permeable AMPAR antagonist) before subsequent training in context B. Injections of CPP did not impair learning in context B while injections of CPP + IEM-1460 produced significant deficits (saline n = 10, CPP n = 10, CPP + IEM-1460 n = 11). (***F***) Wild-type mice were trained in context A and then received injections of saline, CPP or CPP +IEM-1460 before subsequent training in context B. Injections of CPP did not impair learning in context B while injections of CPP + IEM-1460 produced significant deficits (saline n = 8, CPP n = 8, CPP + IEM-1460 n = 8). Error bars represent ± SEM and * indicates statistical significance (p<.05).

Experience-dependent learning can be studied in the hippocampus using the ‘upstairs/downstairs’ procedure. In this task, an initial learning event on day 1 (context A; ‘upstairs’) is NMDAR-dependent, while a subsequent learning event the next day (context B; ‘downstairs’) is NMDAR-independent [5], [6], [7], [8]. We first established the ‘upstairs/downstairs’ effect in wild-type mice using the NMDAR antagonist CPP. 129S6 mice were conditioned sequentially in two different environments (design illustrated in Fig. 8*A*). One group of mice received saline injections before training in context A on day 1 and CPP before training in context B on day 2. A second group of mice received CPP prior to training in context A on day 1, followed by an injection of saline before training in context B on day 2. Both groups of mice were then tested on days 3 and 4 (no injections) to assess memory for each context. Similar to previous reports, we found that injections of CPP given prior to training were more effective at blocking learning in context A than context B (significant context x drug interaction F (1, 14)  = 11.824, p<.05) (Fig. 8*B*) [5], [6], [7], [8]. This demonstrates that NMDAR activation is required for initial learning in context A, but not subsequent learning in context B.

We next studied the effect of the GluR2-cKO on fear learning in the ‘upstairs/downstairs’ paradigm. We found that deletion of GluR2 was more effective at blocking initial learning in context A than subsequent learning in context B (significant context x genotype interaction (F (1, 47)  = 5.802, p<0.05)) (Fig. 8*C*). This suggests that GluR2-cKO mice are more impaired on hippocampus-dependent learning tasks that require the NMDAR (context A; day 1) than tasks that are independent of the NMDAR (context B; day 2).

In the next experiment we verified that GluR2-cKO mice were indeed using an NMDAR-independent learning mechanism in context B (Fig. 8*D*). To do this we used the same behavioral design as in our first experiment (illustrated in Fig. 8*A*). In control animals, we once again found that CPP was more effective at blocking learning in context A than context B (significant context x drug interaction F (1, 35)  = 12.728, p<.05). Similarly, GluR2-cKO mice that received saline were once again impaired in context A, but not context B, relative to control mice that received saline (significant context x genotype interaction, F (1, 21)  = 10.347, p<.05). Lastly, the increased freezing GluR2-cKO mice exhibited in context B was not reduced by the administration of CPP (effect of context, F (1, 18)  = 46.036, P<.05; no context x drug interaction, F<1). These results demonstrate that learning in context B occurs via an NMDAR-independent plasticity mechanism in both control and GluR2-cKO mice. In the next experiments we examined cellular mechanisms that could potentially mediate learning in the absence of NMDAR activation.

Our electrophysiological data indicate that Ca^2+^-permeable AMPARs are present in the CA1 region of our GluR2-cKO mice and are able to mediate LTP in the absence of NMDAR activation. It is possible, therefore, that these receptors contribute to NMDAR-independent learning in context B. To examine this idea we trained GluR2-cKO animals using the ‘upstairs/downstairs’ design. We trained the mice in context A and then administered saline, CPP, or CPP + IEM-1460 (a Ca^2+^-permeable AMPAR antagonist) immediately prior to learning in context B (Fig. 8*E*). Consistent with our previous results, planned comparisons (Fisher's PLSD) showed that blocking NMDARs with CPP did not impair learning in context B relative to saline controls (p>.05). In contrast, the addition of a Ca^2+^-permeable AMPAR antagonist produced a significant memory deficit relative to controls that received saline (p<.05). This data suggests that GluR2-lacking receptors contribute to NMDAR-independent learning in our knockout animals.

In our last experiment we determined if Ca^2+^-permeable AMPARs also contribute to NMDAR-independent learning in wild-type mice (Fig. 8*F*). Planned comparisons (Fisher's PLSD) showed that after training in context A, blocking NMDARs did not affect learning in context B relative to mice that received saline (p>.05). In addition, just as in the GluR2-cKO mice, the addition of IEM-1460 significantly impaired learning in context B (p<.05). This suggests that GluR2-lacking AMPARs play a role in NMDAR-independent learning in wild-type mice. This result is consistent with studies showing that activation of NMDARs can induce the synaptic expression of Ca^2+^-permeable AMPARs [9], [10], [12], [52].

## Discussion

### Impaired NMDAR-dependent learning in GluR2-cKO mice

The current study is the first to demonstrate that conditional deletion of GluR2 in approximately 50% of the CA1 region pyramidal cells of the dorsal hippocampus (and layer III of the overlying cortex) selectively impairs NMDAR-dependent learning. In contrast to mice engineered with constitutive deletions of GluR2 [16] our conditional knockout mice showed no motor, sensory or motivational changes and they exhibited normal acquisition of several hippocampus-independent learning tasks. Furthermore, presence of AMPA receptors lacking GluR2 did not lead to increased seizure vulnerability, confirming previous observations [55]. In addition, cells lacking GluR2 showed c-fos reactivity after fear conditioning, demonstrating that synaptic transmission is intact during learning. Together, our data suggest that the deficits observed in our GluR-cKO mice resulted from impaired learning and not from changes in performance factors.

Our GluR2-cKO animals exhibited enhanced LTP in CA1 pyramidal cells, a fact that is consistent with studies of other types of GluR2 mutant mice [16], [17], [18]. Previous studies also found that the enhanced LTP in GluR2 mutant mice is completely blocked by postsynaptic infusion of calcium chelators, such as BAPTA [18]. Despite enhanced LTP, GluR2-cKO mice showed learning deficits on tasks that are known to be NMDAR-dependent. A possible explanation for this dissociation is that although the early phases of LTP are enhanced in GluR2-cKO mice, alterations in AMPA receptor trafficking due to the absence of GluR2 might disrupt the maintenance of LTP over longer periods of time than those investigated in our experiments. A recent study has found, however, that the long-lasting, protein synthesis-dependent phase of LTP is not disrupted in GluR2 mutant mice [18] and that the c-terminus of AMPA receptor GluR2 subunits does not have a special role in AMPA receptor trafficking [56]. A more likely explanation, therefore, is that learning is impaired because of the unique induction characteristics of LTP in GluR2-cKO mice.

Hebbian LTP requires coincident postsynaptic depolarization and presynaptic glutamate release, is NMDAR-dependent, and is not induced at hyperpolarizing membrane potentials [22], [23] (for detailed discussion see Text S1). In both our GluR2-cKO mice and controls we found that LTP could be induced at depolarizing membrane potentials (Fig. 4*A*), although this LTP was enhanced in GluR2-cKO animals. NMDARs contributed to LTP in both groups since D-APV significantly reduced the amount of potentiation. We also observed that plasticity mediated by Ca^2+^-permeable AMPARs could be induced when the post-synaptic membrane was *hyper*polarized. This form of LTP was only observed in GluR2-cKO mice and not in wild-type controls (Fig. 4*C*). The addition of this second form of plasticity to excitatory neurons may undermine the specificity imparted by Hebbian plasticity. Consistent with this idea, several studies have shown that the presence of GluR2-lacking AMPARs can reduce the specificity and stability of plastic changes mediated by NMDARs [28], [57]. The current study extends these results by demonstrating that the selective addition of AMPAR-mediated plasticity to CA1 pyramidal cells impairs NMDAR-dependent learning.

Some mutant mice with increased hippocampal plasticity show enhanced learning [58] while others exhibit learning deficits [59], [60], [61]. This raises an important question: what is the mechanism of this discrepancy in learning? Why is learning impaired in some mutant animals but not others? Our results suggest that the rules by which plasticity is induced are important for learning and not the magnitude of LTP. In particular, disruption of Hebbian plasticity at CA1 synapses in the hippocampus appears to interfere with NMDAR-dependent memory formation.

### NMDAR-independent learning is not impaired in GluR2-cKO mice

Spatial and contextual memories can be acquired in the absence of NMDAR activation if animals have prior experience on a similar behavioral task (‘upstairs/downstairs’ effect) [5], [6], [7], [8]. This finding challenges the view that NMDARs are essential for all types of learning and suggests that plasticity mechanisms in the hippocampus are modulated by experience.

To examine NMDAR-independent learning we conditioned mice sequentially in two different environments (context A followed by context B). Similar to previous work, we found that learning about the first environment (context A) required NMDAR activation while learning about the second environment (context B) did not [7], [8]. This effect was confirmed in four independent experiments (Fig. 8*B*, 8*D*, 8*E* and 8*F*). Using this design, we then showed that memory for context A was impaired in GluR2-cKO mice while learning in context B remained intact. This suggests that GluR2 deletion in CA1 selectively impairs NMDAR-dependent learning. In addition, we found that blocking Ca^2+^-permeable AMPARs during context B learning significantly impaired memory in both wild-type animals and GluR2-cKO mice. Together, these results suggest that NMDAR-independent learning requires the activation of Ca^2+^-permeable AMPARs.

In sum, our study provides a potential cellular mechanism for NMDAR-independent learning. We showed that deletion of the GluR2 subunit in the CA1 region of the hippocampus makes LTP induction possible in the absence of NMDAR activation. This new form of LTP is mediated by Ca^2+^-permeable AMPARs and is functionally distinct from classical NMDAR-mediated LTP. Behavioral experiments revealed that GluR2 deletion produces significant impairments on hippocampus-dependent learning tasks. However, only NMDAR-mediated memory was disrupted in GluR2-cKO mice. Learning that was independent of NMDARs was not impaired and, in fact, required the activation of Ca^2+^-permeable AMPARs. These results suggest that plasticity mediated by Ca^2+^-permeable AMPARs plays a functional and previously undescribed role in NMDAR-independent learning.

## Materials and Methods

### Ethics Statement

All experiments were approved by the UCLA Animal Research Committee (ARC # 2001-104-23 and 1993-295-53) or the Animal Ethics Committee at the Garvan Institute of Medical Research (AEC# 08/20 and 09/14).

### Subjects

Floxed GluR2 mice in a 129S6 background, were generated as described in Text S1. They were crossed to T29-1 mice that express Cre recombinase under control of the CaM kinase II (CaMKII) promoter [4] that had been backcrossed 15+ generations into the 129S6 background. Subsequently fGluR2/fGluR2;Tg^t29-1/+^ mice were mated to fGluR2 homozygote mice, and littermates from the resulting offspring were used for experiments. Mice were group-housed, given free access to food and water and maintained on a 14∶10 light:dark cycle. Behavioral tests were performed during the light phase of the cycle. All experiments were approved by the UCLA Animal Research Committee (ARC) or by the Animal Ethics Committee at the Garvan Institute of Medical Research.

### *In situ* hybridization

GluR2 exon 11 probes were transcribed from the cloned mouse cDNA in vitro using S-35-UTP (Maxiscript kit, Ambion), and column purified (Qiagen). Hybridized slides were coated with emulsion (Amersham), exposed, developed, and counterstained with Nissl stain. The percentage of GluR2-positive cells was counted independently by two pathologists, blinded to age and genotype. For all GluR2-cKO animals, percentages were calculated from a minimum of three slices per animal and a minimum of three animals per group. For control animals, multiple slices from three animals were counted at the 6 week time point, but from only one control animal at the 8 week time point, as preliminary studies on multiple (n = 3) control animals found no change in percent GluR2-positive cells at three time points over a 3 month period (unpublished observations). Independent assessment by the two pathologists showed agreement. P levels were determined by a 2-tailed Mann-Whitney test. See also [62].

### Immunohistochemistry and Stereology

Immunohistochemical and stereology were undertaken as previously described [19]; see also Text S1.

### Electrophysiology

Previously described techniques for extracellular and whole-cell current-clamp recordings in the in-vitro hippocampal slice preparation were used to record EPSPs evoked by Schaffer collateral fiber stimulation in hippocampal CA1 pyramidal cells [15]. Patch-clamp electrodes were filled with a solution containing (in mM) 122.5 Cs-gluconate, 17.5 CsCl, 10 TEA-Cl, 0.2 EGTA, 10 HEPES, 2.0 Mg-ATP, 0.3 Na-GTP, and 0.1 spermine (pH = 7.2). Picrotoxin (100 µM) was added to the bath solution to block inhibitory synaptic potentials during whole-cell current-clamp recordings. Further detail can be found in Text S1.

### Surgery

Mice were randomly assigned to receive a lesion of the hippocampus or sham surgery. Animals were anesthetized with sodium pentobarbital (65 mg/kg) and mounted in a stereotaxic apparatus (David Kopf Instruments, Tujunga, CA). Small burr holes were drilled above the hippocampus. A 10 µl Hamilton syringe was mounted to the stereotax via a microinjection unit (Model 5000; David Kopf Instruments) and used to deliver all solutions. Excitotoxic lesions were made by infusing NMDA (0.2 µl; 10 mg/ml; Sigma, St. Louis, MO) dissolved in physiological saline (0.9%, pH = 7.4) at each site over a 4-minute period. The drug was allowed to diffuse for 2 minutes following each infusion. Infusions were made at four sites (2 anterior, 2 posterior). The two anterior infusions were made at the following coordinates: 1.3 mm posterior to bregma, ±1 mm lateral to bregma, 2 mm ventral from skull surface. The two posterior DH infusions were made at the following coordinates: 2.1 mm posterior to bregma, ±1.5 mm lateral to bregma, 2 mm ventral from skull surface. Sham controls underwent identical surgical procedures without infusions.

### Kainate-induced seizures

Mice received a single intraperitoneal (i.p) injection of kainic acid (25 mg/kg; Sigma, St. Louis, MO) dissolved in phosphate buffered saline (PBS). Animals were observed for seizure-like behavior for 2–4 hours following the kainic acid injection. Seizure-induced activity was scored according to the Racine scale [63], [64]. Animals were scored as follows: 0 - Normal exploratory behavior and grooming; 1 - Cessation of typical activity (walking, grooming, exploring, sniffing); 2 - Forelimb and/or tail extension, appearance of rigidity; 3 - Automatisms (repetitive scratching, circling, head bobbing); 4 - Forelimb clonus, rearing and falling; 5 - Repetitive rearing and falling and forelimb clonus; 6 - Severe tonic clonic seizures; 7 –Death.

### Fear conditioning methods

In experiments 1–4 (Fig. 5), mice were trained in chambers (30 cm ×24 cm ×21 cm; Med Associates, Inc., St. Albans, VT) with clear polycarbonate front doors, white opaque back walls and aluminum sidewalls. Each chamber had a stainless steel grid floor (36 stainless steel rods, 3 mm diameter, spaced 8 mm center to center) and a metal waste pan underneath. Before training and testing, the chambers were cleaned thoroughly with ethanol (95%) and a thin film was sprayed into the waste pan. Background noise (55 dB) was generated by a HEPA filter. Shock and auditory stimuli were controlled by Med-PC software (MedAssociates, Inc., St. Albans, VT). In each context, a single camera recorded the behavior of animals in four chambers. The freezing response was measured using an automated system as described previously [65].

In experiment 1 (Fig. 5*A*, 5*B* and 5*C*), mice were placed in the training context for 2 minutes before shock (2 s, 0.75 mA) was delivered. Animals received either 1 or 5 shocks (1 minute inter-trial interval (ITI)) and were then tested in the training context immediately, 2 hours or 24 hours later. In experiment 2 (Fig. 5*D*) the mice were trained with 5 unsignaled shocks (0.75 mA, 2 s, 1 minute ITI) and the next day received sham surgery or excitotoxic hippocampus lesions (described above). Seven to ten days after surgery the mice received a 5-minute context test. In experiment 3 (Fig. 5*E*) some of the mice were pre-exposed to the training context for 10 minutes in the absence of shock. The remaining mice stayed in their homecages. The next day all animals received a single 0.75 mA shock (2 s) 5 seconds after placement in the training environment. Thirty seconds later they were removed from the context and returned to their homecages. A 5-minute context test was conducted in the training environment the following day. In experiment 4 (Fig. 5*F*) the mice were trained with 5 whitenoise (30 s, 85 dB) shock (2 s, 0.75 mA) pairings (1 minute ITI). The auditory test was conducted 24 hours later in a novel environment (i.e. same conditioning chambers with a triangular insert (22 cm ×22 cm ×22 cm) and a staggered grid floor located in a different room that was dimly lit and had no background noise). The novel environment was cleaned with Windex (TM). Following a 2-minute baseline period the whitenoise stimulus was presented five times, each separated by a 1-minute ITI. The next day the animals were returned to the training chambers for a 5-minute context test.

In experiments 5–8 (Fig. 8) mice were trained on or off drug in two distinct environments. Context A had curved white plastic walls, was dark, and the internal fans were turned off. It was cleaned with 1% acetic acid diluted in deionized water. In contrast, Context B had no wall inserts and was lit with white light. It was scented with 100% wintergreen cleaning solution and cleaned with 70% isopropyl alcohol. Twenty-four hours before each experiment all animals were handled and restrained to habituate them to IP injections. The NMDA receptor antagonist CPP [66] (Sigma-Aldrich, St. Louis, MI) and the GluR2-lacking AMPA receptor selective blocker IEM-1460 [15], [67], [68], [69] (Tocris Bioscience, Ellisville, MI) were dissolved in filtered 0.9% NaCl and administered via intraperitoneal injections. CPP was administered at a dose of 10 mg/kg, and the CPP-IEM cocktail was given at a dose of 10 mg/kg of CPP and 7.5 mg/kg of IEM-1460. On day 1, animals received an IP injection of either vehicle or drug and 30 minutes later were trained in context A. After an initial 3-minute baseline period animals received three unsignaled footshocks (0.5 mA, 2 s) spaced 20 s apart and were removed 30 s after the last shock. On day 2, animals again received an IP injection of drug or saline and 30-minutes later were trained in context B using the same protocol as day 1. The animals received 5-minute tests on days 3 and 4 (no injections) to assess fear in contexts B and A, respectively.

### Morris watermaze

The watermaze apparatus and procedures have been described previously [39], [70], [71]. Mice were trained to find a visible platform located in a fixed spatial location with 2 trials/day for 5 days. Mice received 2 consecutive trials on each day. Each trial began from a randomly chosen starting position and ended when the mouse found the platform or 60 s had elapsed. The distance traveled (m) to the platform was calculated on each trial and averaged for a single day. On day 6 the mice received a 60 s probe test. On this trial, the platform was removed and the mouse released from a novel starting position. Spatial memory was assessed by comparing the percentage of time spent in the target quadrant relative to the other 3 quadrants.

### Radial arm maze

#### Reference memory version

The radial arm maze apparatus and general procedures have been described previously [70], [72]. Before training, the mice were food-deprived to 85% of their free-feeding body weight. During this time they were pre-exposed to 20-mg rodent chow pellets (Bio-Serve; Frenchtown, NJ) in the same food cups that would be found on the radial maze. Mice were then given 2–3 d of pre-exposure to the center of the maze with 5 chow pellets. Afterwards, mice received 9 d of reference memory training on an eight-arm radial maze [40], [41], [42], [43]. Each day consisted of a single trial with four baited arms. The arms were chosen randomly prior to training and the same four arms were baited on each day. Mice had 5 min to retrieve all pellets. An error was defined as an entry into an unbaited arm. The percentage of correct choices was determined by dividing the number of correct choices by the total number of choices and multiplying by 100. There were 4 correct choices to be made and an infinite number of incorrect choices, since animals were free to re-visit arms as many times as they wanted during the 5 minute session. Therefore, 50% is not chance performance. Previous experiments in our lab have shown that rotating the maze and/or obscuring the cues with a curtain impairs performance on our maze (unpublished data). This suggests that animals do not use intra-maze cues to solve the task. In addition, the maze was constructed with clear Plexiglas and cleaned with 95% alcohol between each phase to reduce the contribution of visual and olfactory intra-maze cues.

#### Win-shift version

Following food deprivation and pre-exposure (as described above) the mice received 9 d of win-shift training on the eight-arm radial maze [40], [41], [42], [44], [45], [70], [72]. Each day consisted of two phases. In the first phase, four randomly selected arms were baited and open. Mice had 5 min to retrieve all pellets. After retrieval of the pellets, the mouse was placed in a holding cage (during which time the maze was cleaned with 95% ethanol) and after a 2-min delay returned for the second phase. During this phase, all eight arms were open and the four previously unbaited arms were now baited. An error was defined as a re-entry into an arm that was baited during the first phase of the experiment. The percentage of correct choices was determined by dividing the number of correct choices by the total number of choices and multiplying by 100. After 9 d of training with a 2-min interphase delay the mice were tested for an additional 5 days with increasing delay lengths (10, 60, 120, 240 and 480 min).

### Statistical analysis

Unless otherwise stated, data sets were tested for normality using the Shapiro-Wilks test, or Kolmogorov-Smirnov. If the data were non-normally distributed then they were analyzed non-parametrically. In the behavioral studies, most data were analyzed with an ANOVA and post-hoc comparisons were conducted with Fisher's PLSD. In experiments with specific predictions based on previous data, a priori planned contrasts were used and analyzed using Fisher's PLSD.

## Supporting Information

Text S1 Supplementary Results, Methods, Figures and Table
(0.17 MB DOC)

Figure S1 (A1) The GluR2 flox-neo allele. Shown are exons 10, 11 and 12, the loxP sites (open arrows), neomycin resistance (NeoR) gene, targeting construct (indicated by solid line), and 5′, 3′ and INT probes used for Southern blotting (gray boxes). Exon 11 encodes membrane domains 1 and 2 of the GluR2 protein, including the critical site of RNA editing (Q/R site). B = BamHI, S = SpeI. (A2) The floxed GluR2 (fGluR2) allele. Cre transfection of ES cells containing the GluR2 flox-Neo allele results in excision of the NeoR gene in some ES cells. These cells were then used to generate fGluR2 mice. (A3) The GluR2 knockout (GluR2-KO) allele. In other ES cells, transfected cre excises the exon 11-containing fragment and NeoR gene entirely from the GluR2 gene, leaving a single loxP and BamHI site in its place. These ES cells were used to generate GluR2-KO mice. (B) GluR2 RNA is expressed normally in fGluR2 but not in GluR2-KO mice. The RNAse protection assay (RPA) confirms loss of GluR2 mRNA in the GluR2-KO which was generated by cre recombinase-mediated excision of exon 11, and reveals that hippocampal GluR2 mRNA expression is normal in the WT and homozygous fGluR2 animals. The upper band (lanes 1 and 2) is the protected antisense 32P-labelled RPA probe, while the lower band (also observed in lanes 3 and 4) is an actin antisense probe included as an internal standard. The sense control probe (lane 4) is also shown. A non-RNAse-treated sample was analyzed in parallel for each probe to confirm the expected size reduction of the protected species due to removal of the 5′ and 3′ overhangs of the RPA probe (not shown). (C) Normal GluR2 protein expression in fGluR2 mice but not GluR2-KO mice. Immunoblotting was used to detect the presence of GluR2 in the hippocampus, using an antibody directed against either the N'-terminal or C'-terminal region of the GluR2 protein. 10 ug membrane protein was analyzed in each case. Note that for both antibodies, no GluR2 band is seen when the primary antibody is omitted (no 1′). The upper band indicates non-specific cross reactivity of the secondary antibody, as it is present in the wild-type extracts in which 1′ is omitted. (D) RNA editing is not altered in the fGluR2 mice. An RNA editing assay similar to that previously described (Vissel et al., 2001) was used to assess the proportion of GluR2 or GuR5 mRNA edited at the Q/R editing site. Total mouse hippocampal mRNA or genomic DNA was isolated from wild-type (+/+), homozygote floxed (f/f) or GluR2-KO (−/−) mice. Primer extension yields a 21-mer product if the template is unedited, whereas edited template yields a 24-mer. The intensity ratio between the two bands represents the ratio of unedited to edited mRNA. As expected, there was no evidence of the unedited product in wildtype (+/+) or homozygous fGluR2 mice (f/f), while, as expected, there was no evidence of any GluR2 RNA in the GluR2-KO (−/−) mice. RNA editing at the GluR5 Q/R site also appeared to be unaltered in homozygous fGluR2 mice (f/f) and in GluR2-KO (−/−) mice when compared to wild-type (+/+) mice, as the ratio of the 21-mer to 24-mer product is unchanged. (E) Southern blots demonstrating successful insertion of the construct at the targeted site. A 12.5 Kb BamHI fragment and a 5.5 Kb SpeI fragment are detected using the 5′ probe in DNA from the wild type (+/+) mouse (lanes 1, 2) and in one allele from ES cells heterozygous (+/fNeo) for the flox-Neo cassette (lanes 7, 8). Insertion of the loxP cassette creates a 5′ BamHI restriction fragment of 5 Kb in DNA from mice homozygous (f/f) for the floxed allele, in mice homozygous (−/−) for the GluR2-KO allele and in one allele of DNA from ES cells heterozygous (+/fNeo) for the flox-Neo cassette (lanes 3, 5, 7). Also as expected, the SpeI fragment detected by the 5′ probe remains essentially unchanged in size in DNA from mice homozygous (f/f) for the fGluR2 allele (lane 4), is 3.5 Kb size in DNA from GluR2-KO (−/−) mice (lane 6), and is 7.2 Kb in DNA from ES cells heterozygous (+/fNeo) for the flox-Neo cassette (lane 8). Meanwhile, as expected, the BamH1 fragment detected by the 3′ probe is 12.5 kB in wildtype (+/+) mice (lane 9), and is approximately 6 Kb in DNA from mice homozygous (f/f) for the fGluR2 allele (lane 10), and in mice homozygous (−/−) for the GluR2-KO allele (lane 11). The INT probe detected BamHI and SpeI restriction fragments of the predicted sizes. Importantly, a probe against the NeoR gene detects the expected SpeI restriction fragment in ES cells heterozygous for the flox-Neo cassette (lane 20) but not in DNA isolated from mice heterozygous for the fGluR2 allele (lane 19).
(1.84 MB TIF)

Figure S2 Relative gene expression of NMDA and non-NMDA receptor subunits in GluR2-KO mice (n = 5) and their littermate controls (n = 6), normalized against GAPDH. Knock-out of GluR2 did not alter gene expression of NMDA or non-NMDA receptor subunits.
(0.49 MB TIF)

Figure S3 (A) The GluR2-lacking AMPAR blocker IEM1460 inhibits synaptic transmission in the CA1 region of hippocampal slices from GluR2-cKO mice (n = 3) and global GluR2 KO mice (triangles, n = 3) but has no effect on transmission in control slices (gray symbols, n = 3). Bath application of IEM1460 (100–200 Î¼M) is indicated by the bar. The histograms at right show the mean (Â±SEM) change in synaptic transmission present at the end of a 30-minute application of IEM1460 in slices from wild type (gray bar), GluR2-cKO (black bar), and global GluR2 KO mice (open bar, *p<0.001 compared to control). (B) The input/output function for basal synaptic transmission was generated by comparing fiber volley amplitudes and fEPSP slope for fEPSPs evoked using stimulation intensities corresponding to 25, 50, 75, and 100% of the maximal fEPSP amplitude. Note that the input/output function in slices from GluR2-cKO mice (black, n = 6) is shifted to the right compared to control slices (gray, n = 6). Fiber volley/EPSP slope ratios in slices from GluR2-cKO mice were significantly different from control slices at all stimulation intensities tested (* p<0.005). (C) Paired-pulsed facilitation in control and GluR2-cKO slices. Pairs of presynaptic fiber stimulation pulses were delivered with inter-pulse intervals from 25 to 200 milliseconds and the ratio was calculated as the slope of the 2nd response/1st response X 100. Although paired-pulse facilitation tends to be large in slices from GluR2-cKO mice (black, n = 5) compared to control slices (gray, n = 5), a significant enhancement was seen only at the 100 millisecond interval (*p<0.02).
(0.45 MB TIF)

Figure S4 Error bars represent Â± SEM and * indicates statistical significance (p<.05). (A) Controls (n = 9) and GluR2-cKO (n = 14) showed similar levels of activity on the openfield. (B) Motor learning on the Rotarod was equivalent in controls (n = 9) and GluR2-cKO (n = 14) mice. (C) The unconditioned response to shock was examined by calculating the velocity (cm/s) of each animal during footshock. Data for the 1 (controls n = 13, GluR2-cKO n = 13) and 5 (controls n = 21, GluR2-cKO n = 12) shock groups were combined for the graph. Controls and GluR2-cKO mice showed equivalent increases in velocity during shock relative to baseline. (D) The minimum (gray) and maximum (black) extent of excitotoxic dorsal hippocampus lesions in controls (n = 6) and GluR2-cKO mice (n = 9).
(0.65 MB TIF)

Table S1 Standard curves and melt curves are shown for qRT-PCR analysis of gene expression of non-NMDA receptor subunits (GluR 1, GluR2, GluR3, GluR4, GluR5, GluR6), NMDA receptor subunits (NR1, NR2A), and the calibrator gene (GAPDH).
(2.20 MB TIF)
