# Supplementary material for: A Role for Calcium-Permeable AMPA Receptors in Synaptic Plasticity and Learning
Source: PLoS One. 2010 Sep 29;5(9):e12818. doi: 10.1371/journal.pone.0012818 (PMC2947514; doi:10.1371/journal.pone.0012818)
Supplement: Text S1 — Supplementary Results, Methods, Figures and Table (0.17 MB DOC) [file pone.0012818.s001.doc]

Supplementary Information

Supplementary Results, Methods, Figures and Table

**Calcium-permeable AMPA receptors contribute to learning and plasticity and in the hippocampus.**

Brian J Wiltgen, Gordon Royle, Erin E. Gray, A. Abdipranoto, Nopporn Thangthaeng, Nate Jacobs, Faysal Saab, Susumu Tonegawa, Thomas J O’Dell, Stephen F Heinemann, Michael S Fanselow, and Bryce Vissel.

**Supplementary Information: Results**

##### Generation of floxed GluR2 mice

To study the effects of calcium-permeable AMPARs on plasticity and memory we generated a line of mice in which GluR2 was deleted from pyramidal cells of the hippocampus. We engineered loxP sites in the introns that surround exon 11 of the GluR2 (Gria2) gene. Exon 11 encodes membrane domains 1 and 2 of the mature GluR2 protein and is essential for gene function ((Dingledine et al., 1999)). Our strategy for generating a floxed GluR2 (controls; fGluR2) mouse line is shown in Fig. S1 and is similar to that previously reported (Vissel et al., 2001).

In order to ablate GluR2 without affecting sensory and motor function, homozygous floxed mice (maintained in a 129S6 background) were crossed with T29-1 Cre transgenic mice which had been backcrossed more than fifteen generations to 129S6. This strategy ensured that the mice we studied were in a pure 129S6 background. T29-1 mice express Cre recombinase under control of the Cam Kinase II (CamKII) promoter (Tsien et al., 1996b; Tsien et al., 1996a). Therefore homozygous fGluR2 mice that are also heterozygous for the T29-1 Cre recombinase transgene (which we refer to as GluR2-cKO mice) are expected to contain a conditional knockout of GluR2 in the forebrain.

In addition, we generated a complete GluR2 knockout mouse line (GluR2-KO) by mating fGluR2 mice to mice expressing Cre recombinase in sperm (Vissel et al., 2001) (allele shown in Fig. S1*A*(3)). Southern blot analyses confirmed that the Gria2 gene was indeed altered as predicted in the fGluR2 and GluR2-KO mice (Fig. S1*E*).

#### *Analysis of the mice*

We confirmed that the floxed allele (present in control mice) is expressed normally and, also, that Cre-mediated recombination between loxP sites leads to a complete ablation of GluR2 expression. We utilized RNAse protection assays to assess GluR2 mRNA expression, and immunoblotting to assess GluR2 protein expression, both shown in Fig. S1. As expected, GluR2 mRNA (Fig. S1*B*) and protein expression (Fig. S1*C*) were normal in the floxed mice compared to wild-type mice, but GluR2 expression was abolished in the GluR2-KO mice. Furthermore, no truncated GluR2 protein was detected in immunoblots using an antibody directed against the N-terminus of GluR2, even on shorter gel runs. Consistent with previously published results (Jia et al., 1996) we found that deletion of the GluR2 subunit did not affect the expression of other glutamate receptor subunits in these GluR2-KO mice (Fig. S2). These results are identical to those of Jia et al, who also demonstrated that their GluR2 knockout mice show no change in expression of glutamate receptor subunits (Jia et al., 1996).

As shown in Fig. 1, we demonstrate a selective knockout of GluR2 chiefly in the CA1 region of the dorsal hippocampus and Cortex layer III, in GluR2-cKO mice age 6-8 weeks. Clearly, as with all studies using transgenic mice expressing Cre recombinase, we cannot exclude deletions of GluR2 in areas we have not visualized in our in situ hybridization experiments, however this does not affect our interpretation that calcium-permeable AMPARs can contribute to learning. In particular, as shown later in this study, mice engineered with GluR2-lacking AMPARs show normal second learning, unless an antagonist of calcium-permeable AMPARs is administered (Fig. 8). We also describe in our study that, unlike GluR2-KO mice, the GluR2-cKO mice demonstrate a deficit in tasks normally associated with the hippocampus and not in other tasks, suggesting that the knockout is selective to the hippocampus (Figs 5-7).

GluR2, GluR5 and GluR6 pre-mRNA is subject to RNA editing, which converts a CAG codon for glutamine (Glu; Q) to a CGG codon for arginine (Arg; R), in the mRNA. Complete Q/R site RNA editing is essential for normal GluR2 gene function (Seeburg and Hartner, 2003) while about 50% of GluR5 mRNA is edited to R in the mouse hippocampus (Vissel et al., 2001). In order to confirm that the loxP sites in the GluR2 gene do not interfere with RNA editing of GluR2, we used an RNA editing assay. As expected no unedited GluR2 was observed as a result of the loxP sites present in the intron of the GluR2 gene. We also observed that the percent of GluR5 editing appeared unchanged in floxed mice and even in GluR2-KO mice (Fig. S1*D*).

Our combined results demonstrate that the loxP sites we engineered into the GluR2 gene do not affect the level of GluR2 expression or Q/R site RNA editing and, further, that Cre recombinase-mediated recombination between loxP sites abolishes GluR2 gene function. Therefore we conclude that the loxP sites we engineered in the GluR2 gene do not interfere with GluR2 gene function and that Cre recombinase expression mediates a complete knockout of the GluR2 subunit in cells where Cre recombinase is expressed.

**Gene expression of non-NMDA and NMDA receptor subunits.**

Gene expression levels of non-NMDA and NMDA receptor subunits were quantitated by qPCR and normalized against GAPDH. Analysis of gene expression showed that, as expected, there is no GluR2 expression in the GluR2 knockout mouse. Moreover when comparing the global GluR2 knock-out and their littermate controls, there is no significant difference between any of the non-NMDA receptor subunits and NMDA subunits (Fig. S2). These findings are in agreement with those previously reported (Jia et al., 1996).

**Definition of Hebbian Plasticity**

Throughout our manuscript we have used the term Hebbian plasticity to refer to that plasticity which is contingent upon the concurrent release of neurotransmitter and post-synaptic membrane depolarization. This usage ultimately derives from the definition originally proposed by Donald Hebb: *“When an axon of cell A is near enough to excite cell B or repeatedly or persistently takes part in firing it, some growth process or metabolic change takes place in one or both cells such that A's efficiency, as one of the cells firing B, is increased.” (Hebb, 1949)*. Other authors have used the term anti-Hebbian plasticity to refer to plasticity induced when the post-synaptic membrane is hyperpolarized (Lamsa et al., 2007; Kullmann and Lamsa, 2008).

NMDAR-mediated plasticity cannot be induced at hyperpolarizing postsynaptic membrane potentials. This is consistent with the I/V relations of NMDA receptors, which predict that these receptors will flux calcium the most strongly when the post-synaptic membrane is depolarized. Conversely, the I/V relations of GluR2-lacking AMPA receptors demonstrate that these receptors will carry increasing inward current (carried in part by influx of Ca^2+^ ions) as the post-synaptic membrane potential becomes increasingly negative. Note however that GluR2-lacking AMPARs still flux some current at depolarizing (but still negative) membrane potentials – this is illustrated nicely in the work of Toth and McBain (Toth and McBain, 1998). In the hippocampal CA1 region of GluR2-cKO mice, we observed that plasticity could as expected be induced at both depolarizing and hyperpolarizing post synaptic membrane potentials in cells where NMDARs and GluR2-lacking AMPARs are present (Fig 4). Our conclusion is that while LTP is enhanced in GluR2-cKO mice (Fig 3), the specificity associated with NMDAR-dependent LTP has been lost in these mice.

**Loss of GluR2 produces the expected synaptic effects**

We confirmed that the absence of GluR2 at synapses resulted in the expected effect on AMPA receptor-mediated synaptic transmission. First, we used fEPSP recordings to confirm that the sensitivity of synaptic transmission to blockers of GluR2-lacking AMPARs, was changed as expected. As shown in Fig. S3, bath application of a high concentration (100-200 μM) of the selective GluR2-lacking AMPAR blocker IEM1460 inhibited synaptic transmission in the CA1 region of GluR2-cKO mice to 64 ± 4% of baseline (n = 3) compared to 98 ± 4% of baseline in control slices (n = 3). This is consistent with our in situ hybridization analysis of GluR2 expression, showing that overall GluR2 expression in the hippocampal CA1 region is reduced by approximately 50% in the GluR2-cKO mice (Fig. 1). As expected, the inhibition of synaptic transmission in cells from Global GluR2 KO mice (in which all neurons lack GluR2) by IEM1460 was even more profound, reducing transmission to 21 ± 2% of baseline (n = 3) (Fig. S3*A*). We then confirmed that input/output functions and paired-pulse facilitation of synaptic transmission in GluR2 mutant mice was altered as expected. Indeed, similar to findings from previous studies of both global GluR2 knockouts (Jia et al., 1996) and forebrain-specific GluR2 mutants (Shimshek et al., 2006), input/output curves were right-shifted in the mutants compared to controls (Fig. S3*B*). Finally, as expected (Jia et al., 1996; Shimshek et al., 2006), there was relatively little effect of the GluR2 deletion on paired-pulse facilitation (Fig. S3*C*).

**Motor function and pain processing in GluR2-cKO mice**

We analyzed motor function in GluR2-cKO mice because deletion of this subunit in the forebrain has been shown to alter exploration and coordination (Gerlai et al., 1998; Shimshek et al., 2006). We found that selective deletion of GluR2 in CA1 and adjacent cortical regions did not affect activity levels in the openfield (no effect of genotype F (1, 21) = 1.03, p > .05) or motor learning on the rotarod (no effect of genotype F (1, 21) = 1.97, p > .05) (Fig. S4*A* and S4*B*). This suggests that changes in exploration and coordination result from deletion of GluR2 in brain regions that are not affected in our conditional knockout mice.

To ensure that our mutation did not affect shock reactivity, we examined each animal’s velocity (activity burst) during shock and an equivalent baseline period. Velocity (cm/s) was measured because we have found it to be a very sensitive indicator of shock reactivity ((Godsil et al., 1997)). Both controls and knockout mice showed a significant increase in velocity during shock (main effect of period (baseline vs. shock) F (1, 55) = 335.95, p < .05) that did not differ between groups (no period x genotype interaction F (1, 55) = 2.943, p > .05) (Fig. S4*C*).

**Post-shock freezing as a measure of short-term memory**

Freezing following shock is completely a conditional response (CR) to contextual cues and in no part an unconditional response (UR) to the shock. While freezing is the most studied behavior in this respect, data on other fear responses (startle, defecation, analgesia) all follow the same principles. To briefly summarize some of the key literature on this issue: sufficient context change after shock eliminates the freezing response even when you are looking at the post-shock period (Fanselow, 1980; Fanselow, 1982, 1984; Fanselow, 1990). Delaying the time between shock and test, which should allow a UR to dissipate, has no effect on freezing (Fanselow, 1980; Fanselow, 1982, 1984). Preventing context freezing prior to shock completely eliminates post-shock freezing (Fanselow, 1986, 1990). Massing USs leads to reduced post-shock freezing, again an opposite result from what is expected if freezing was a UR (Fanselow and Tighe, 1988; Fanselow et al., 1993). These data clearly demonstrate that post-shock freezing is a CR to contextual cues.

**Auditory fear conditioning in GluR2-cKO mice**

The unconditioned freezing response to the first whitenoise presentation (before shock was delivered) was very low in both groups (controls = 14%, GluR2-cKOs = 9%). After auditory conditioning, freezing to the whitenoise was significantly higher (test session, controls = 65%, GluR2-cKOs = 56%). These data clearly demonstrate that freezing to the whitenoise stimulus was a learned response. In addition, a direct comparison of auditory freezing levels during training and testing revealed a significant increase in tone fear that did not interact with genotype (main effect of session F (1, 31) = 200, p < .05) (no session x genotype interaction F < 1). Similar to our analyses reported in the manuscript, this indicates that the amount of auditory fear acquired by controls and GluR2-cKO mice was equivalent. Moreover, a direct comparison of auditory freezing during the test revealed that controls and GluR2-cKO mice were not significantly different (p > .05).

**Supplementary Information: Methods**

**Generation and breeding of a GluR2 regional knockout mouse.**

DNA cloned from a 129S6 DNA genomic library was used to generate a targeting construct. Following electroporation into ES cells derived from 129S6 mice (Robertson et al., 1986) G418-resistant colonies were isolated and analyzed by Southern blot. ES cells containing allele-1 shown in Fig. S1*A* were then selected and electroporated with a cre-expressing plasmid as described (pOG231, see ref (O'Gorman et al., 1997)). ES cell colonies were then grown in the absence of G418, and ES cell colonies containing the allele shown in Fig. S1*A*(2) were expanded for blastocyst injection. The resulting chimeric founder mice were bred to 129S6 wildtype mice generating 129S6 isogenic mice heterozygous for the floxed allele. The mice were characterized by Southern blotting (Fig. S1*E*) and bred to homozygosity. Homozygous fGluR2 mice were periodically crossed to 129S6 mice from Taconic to ensure they remain isogenic. fGluR2 homozygote mice were bred with T29-1 (Tg^t29-1/+^) mice and heterozygous fGluR2/+;Tg^T29-1^ offspring were bred with fGluR2 homozygote mice to generate homozygous fGluR2/fGluR2;Tg^t29-1/+^ mice. Subsequently, these homozygous fGluR2/fGluR2;Tg^t29-1/+^ mice were mated to fGluR2 homozygote mice, and littermates from the resulting offspring were used for experiments. Genotyping was performed by PCR.

**SDS-PAGE and immunoblot analysis.**

Proteins were separated on SDS-PAGE and transferred to Immobilon-P membranes and treated as described (Sans et al., 2003; Abdipranoto-Cowley et al., 2009). Images were produced by exposure to chemiluminescence.

**RNAse protection assay.**

Templates for synthesizing probe were provided for actin (RPA-III kit; Ambion) and were generated for GluR2 exon 11 from cloned mouse cDNA (gift from M.Mishina). RPA probes were transcribed in vitro with a-^32^P-UTP (Maxiscript kit, Ambion). Approximately 5 µg of hippocampal total RNA was used in the protection assays (RPA-III kit; Ambion). Protected products were quantified by densitometry on phosphorimager images (Imagequant NT; Molecular Dynamics, Sunnyvale, CA). All results were normalized to actin controls. The ratio of intensity between the two bands corresponds to the expression level of GluR2. See also (Vissel et al., 2001).

**Editing Assay.**

A cycled primer extension editing assay was used (Vissel et al., 2001). Total RNA was isolated from hippocampi or whole brains (TRIZOL; Life Technologies, Rockville, MD). cDNA was synthesized (Thermoscript; Life Technologies), and DNA amplified across the editing site prior to the cycled primer extension assay. Bands were quantified by densitometry on phosphorimager images (Imagequant NT; Molecular Dynamics, Sunnyvale, CA). See also (Vissel et al., 2001).

**Tissue preparation for immunohistochemistry**

Mice were perfused transcardially with 0.9% saline followed by 4% paraformaldehyde (in PBS) at a rate of 10 ml/min for 10 minutes. Brains were harvested and post-fixed in 4% paraformaldehyde (in PBS) for 2 hours then cryoprotected in 30% sucrose (in PBS) for 2 days. Tissue was blocked in PCT and frozen at -80°C. Tissue was sectioned at 40 μm using a cryostat and stored at 4°C. See also (Vissel et al., 2001; Abdipranoto-Cowley et al., 2009).

**Immunohistochemistry**

For fluorescent immunohistochemistry, tissue sections were incubated in anti-cfos or anti-GluR2 (1:500), washed, then incubated in anti-rabbit Alexa Fluor 568 or 488 (1:500). Tissue sections were washed, incubated in DAPI (1:1000) at RT for 10 minutes, washed, mounted, and coverslipped, see (Abdipranoto-Cowley et al., 2009).

For neuronal population estimates, non-fluorescent immunohistochemistry was performed using biotinylated secondary antibodies, biotin-avidin peroxidase complex and diaminobenzidine. Tissue sections were incubated in mouse anti-NeuN (1:500) at 4°C for 3 days, washed 3x with PBS, then incubated in biotinylated anti-mouse antibody (1:250) overnight. Sections were incubated in biotin-avidin peroxidase complex, washed with PBS, visualized with DAB for 5 minutes at RT, mounted and coverslipped.

**Stereology**

Our approach to counting neurons followed the protocols we have previously described (Abdipranoto-Cowley et al., 2009). Briefly, every sixth section was prepared from coronal brain slices. A total of 5 sections were sampled with antero-posterior positions from bregma of -1.34, -1.58, -1.82, -2.06, and -2.3 mm. The total neuronal population was estimated in the DG, CA3 and CA1 using the optical fractionator probe in Stereo Investigator 7 (Microbrightfield). Design parameters were determined prior to commencement of the study. A minimum 5 sampling sites were sampled per section on a grid size of 240 um x 110 um. At each sampling site a counting frame size of 30 um x 30 um, guard zones of 5 um and a dissector height of 10 um were used.  The DG was defined as the region including the granular cell layer and the hilar.

The number of cells that were immunopositive for more than one cell marker in defined regions of the hippocampus was determined using unbiased stereology and confocal microscopy at 40x magnification. The entire region of interest was sampled. Guard zones of 5 μm and a dissector height of 10 μm were used. The number of particles sampled was converted into the total number of particles in the region of interest using the optical fractionator design method.

where:

N is the total number of particles; *bsf* is the block sampling fraction; *ssf* is the section sampling fraction; *hsf* is the height sampling fraction; *h* is the constant height of the optical dissectors; *t* is the local section thickness; ∑ Q^-^ is the total count of particles finally sampled by the optical dissectors

**Histology**

Histological verification of the lesion locations was performed at the end of behavioral testing. Mice were perfused transcardially with 0.9% saline followed by 10% formalin. Following extraction from the skull, the brains were post-fixed in 10% formalin overnight and then stored in 30% sucrose until sectioning. Coronal sections (40 μm thick, taken every 120 μm) were cut on a cryostat (-16°C) and mounted on slides. After drying, the sections were stained with 0.25% thionin to identify neuronal cell bodies. Lesions were verified by visual inspection of the stained sections reconstructed on the rat brain atlas (Paxinos and Watson, 1998).

**Image acquisition**

All images were obtained using Leica DM IRE2 TCS SP2 AOBS inverted laser scanning confocal. Images were taken at 63x and confocal z stack images were obtained using 100x with 4x optical zoom. Images were compiled and analyzed using Adobe Photoshop CS2.

**RT-PCR and qPCR analysis of non-NMDA and NMDA receptor subunit gene expression**

Brain regions were dissected from either GluR2-KO mice or their littermate controls, all aged 8 weeks old. Each sample was homogenized in Trizol (Invitrogen, Victoria, Australia) by passing through a 25 gauge needle. Extraction of total RNA was then carried out according to manufacturer’s instructions with addition of the optional step to remove lipid and large genomic DNA. Isolated RNA was then treated with Turbo DNase (Ambion, Victoria, Australia) to remove any contaminating traces of genomic DNA. After DNase inactivation, 1 ug of total RNA from each sample was converted to cDNA using oligodT and SuperScript III reverse transcriptase (Invitrogen, Victoria, Australia) according to manufacturer’s instructions. Using ABI 7900 (Applied Biosystem, Victoria, Australia), qPCR was performed on the newly generated cDNA, using SensiMixdT (Quantace, New South Wales, Australia) and Syto 9 (Invitrogen, Victoria, Australia). The PCR protocol are as followed: 50°C for 2 min, 95°C for 10 min, 95°C for 10 sec, 65°C for 1min (repeat for 40 cycles), then follow by melt curve analysis for GluR2, GluR5, NR1, NR2A, and NR2B. The same protocol was used for GluR1, GluR3, GluR4, GluR6, except the annealing temperature was lowered to 60°C. GAPDH was run at both temperatures and was used for normalization at each set of annealing temperatures.

**Openfield**

The activity monitors (Med-Associates Inc., St. Albans, VT) were made of polycarbonate plastic (27 x 27 x 20.3 cm). Activity was tracked by 16 photobeams spaced evenly apart on both x- and y-axes. Mice were placed in the chambers located in a dimly lit room and ambulatory counts were calculated over a 20-min period. Background noise (55dB) was generated by a HEPA filter. The chambers were washed with Windex^TM^ between squads.

**Rotarod**

Mice were placed on the Rotarod (TSE Systems, Germany) while it was rotating at 5 rpm. As soon as all mice were on the apparatus, the acceleration and timer were activated and the rotational speed steadily increased from 5 to 60 rpm over the course of 3 min. On each day, every mouse was tested three times sequentially and the results averaged.

**Surgery**

Mice were randomly assigned to receive a lesion of the hippocampus or sham surgery. Animals were anesthetized with sodium pentobarbital (65 mg/kg) and mounted in a stereotaxic apparatus (David Kopf Instruments, Tujunga, CA). Small burr holes were drilled. A 10 ul Hamilton syringe was mounted to the stereotax via a microinjection unit (Model 5000; David Kopf Instruments) and used to deliver all solutions. Excitotoxic lesions were made by infusing NMDA (0.2 μl; 10 mg/ml; Sigma, St. Louis, MO) dissolved in physiological saline (0.9%, pH = 7.4) at each site over a 4-minute period. The drug was allowed to diffuse for 2 minutes following each infusion. Infusions were made at four sites (2 anterior, 2 posterior). The two anterior infusions were made at the following coordinates: 1.3 mm posterior to bregma, ± 1 mm lateral to bregma, 2 mm ventral from skull surface. The two posterior DH infusions were made at the following coordinates: 2.1 mm posterior to bregma, ± 1.5 mm lateral to bregma, 2 mm ventral from skull surface. Sham controls underwent identical surgical procedures without infusions.

**Detailed electrophysiology methods**

Hippocampal slices (400 μm thick) were prepared using standard techniques and maintained in interface-type chambers constantly perfused (at 2-3 mL/min) with an oxygenated (95% O_2_/5% CO_2_) artificial cerebrospinal fluid (ACSF) containing 124 mM NaCl, 4.4 mM KCl, 25 mM Na_2_HCO_3_, 1.0 mM NaH_2_PO_4_, 1.2 MgSO_4_, 2.0 mM CaCl_2_, and 10 mM glucose ((Gray et al., 2007)). In experiments using extracellular recordings, field EPSPs (fEPSPs) evoked by a bipolar stimulating electrode placed in stratum radiatum were recorded using a glass, ACSF-filled microelectrode placed in stratum radiatum (resistance = 5 – 10 Mohms). The basal stimulation rate was 0.02 Hz and the intensity of presynaptic fiber stimulation was adjusted to evoke fEPSPs with amplitudes corresponding to 50% of the maximal fEPSP amplitudes evoked by strong pulses of presynaptic fiber stimulation in each slice.

In whole-cell current-clamp recordings, slices with the CA3 region removed were bathed in a modified ACSF containing elevated concentrations of CaCl_2_ and MgSO4 (4.0 mM each), a lower concentration of KCl (2.2 mM), and 100 μM pictrotoxin was added to block inhibitory synaptic transmission. Low resistance (3 – 5 Mohm) patch-clamp electrodes were filled with a solution containing 122.5 mM Cs-gluconate, 17.5 mM CsCl, 10 mM TEA-Cl, 0.2 mM EGTA, 10 mM HEPES, 2.0 mM Mg-ATP, 0.3 mM GTP, and 0.1 mM spermine (pH = 7.2). The intensity of presynaptic fiber was adjusted to evoke EPSPs with amplitudes between 5 and 10 mV (basal stimulation rate = 0.05 Hz). Constant injections of hyperpolarizing current was used to maintain membrane potentials at -80 to -85 mV and a 50 millisecond-long, 0.1 nA pulse of hyperpolarizing was injected 170 milliseconds after each EPSP to monitor input and access resistance throughout the experiment. The pairing protocol used to induce LTP in these experiments (100 pulses of 10 Hz presynaptic fiber stimulation paired with depolarization or hyperpolarization of the postsynaptic cell) was delivered within 20 minutes of whole-cell recording to avoid wash-out of LTP that can occur during whole-cell recordings. Long-term depression was examined using slices bathed in ACSF containing 4.0 mM CaCl_2_ and maintained in submerged-slice recording chambers.

**Supplementary References:**

Abdipranoto-Cowley A, Park JS, Croucher D, Daniel J, Henshall S, Galbraith S, Mervin K, Vissel B (2009) Activin A is essential for neurogenesis following neurodegeneration. Stem Cells 27:1330-1346.

Dingledine R, Borges K, Bowie D, Traynelis SF (1999) The glutamate receptor ion channels. Pharmacol Rev 51:7-61.

Fanselow MS (1980) Conditional and unconditional components of post-shock freezing. Integrative Psychological and Behavioral Science 15:177-182.

Fanselow MS (1982) The postshock activity burst. Animal Learning & Behavior 10:448-454.

Fanselow MS (1984) Opiate modulation of the active and inactive components of the postshock reaction: parallels between naloxone pretreatment and shock intensity. Behav Neurosci 98:269-277.

Fanselow MS (1986) Associative vs topographical accounts of the immediate shock-freezing deficit in rats: implications for the response selection rules governing species-specific defensive reactions. Learn Motiv:16-39.

Fanselow MS (1990) Factors governing one-trial contextual conditioning. Anim Learn Behav:264–270.

Fanselow MS, Tighe TJ (1988) Contextual conditioning with massed versus distributed unconditional stimuli. Journal of Experimental Psychology: Animal Behavior Processes 14:187-199.

Fanselow MS, DeCola JP, Young SL (1993) Mechanisms responsible for reduced conditioning with massed unsignaled unconditional stimuli. Journal of Experimental Psychology: Animal Behavior Processes 19:121-137.

Gerlai R, Henderson JT, Roder JC, Jia Z (1998) Multiple behavioral anomalies in GluR2 mutant mice exhibiting enhanced LTP. Behav Brain Res 95:37-45.

Godsil BP, Spooner JR, Anagnostaras SG, Gale GD, Fanselow MS (1997) Quantification of the unconditional response to footshock in the rat: measurement of the activity burst with the use of computer-based image analysis. In: Society for Neuroscience, New Orleans, LA:1612.

Gray EE, Fink AE, Sarinana J, Vissel B, O'Dell TJ (2007) Long-term potentiation in the hippocampal CA1 region does not require insertion and activation of GluR2-lacking AMPA receptors. J Neurophysiol 98:2488-2492.

Hebb DO (1949) The Organization of Behavior. New York: John Wiley & Sons.

Jia Z, Agopyan N, Miu P, Xiong Z, Henderson J, Gerlai R, Taverna FA, Velumian A, MacDonald J, Carlen P, Abramow-Newerly W, Roder J (1996) Enhanced LTP in mice deficient in the AMPA receptor GluR2. Neuron 17:945-956.

Kullmann DM, Lamsa K (2008) Roles of distinct glutamate receptors in induction of anti-Hebbian long-term potentiation. J Physiol 586:1481-1486.

Lamsa KP, Heeroma JH, Somogyi P, Rusakov DA, Kullmann DM (2007) Anti-Hebbian long-term potentiation in the hippocampal feedback inhibitory circuit. Science 315:1262-1266.

O'Gorman S, Dagenais NA, Qian M, Marchuk Y (1997) Protamine-Cre recombinase transgenes efficiently recombine target sequences in the male germ line of mice, but not in embryonic stem cells. Proc Natl Acad Sci U S A 94:14602-14607.

Paxinos G, Watson C (1998) The rat brain in stereotaxic coordinates, 4th edition. New York: Academic Press.

Robertson E, Bradley A, Kuehn M, Evans M (1986) Germ-line transmission of genes introduced into cultured pluripotential cells by retroviral vector. Nature 323:445-448.

Sans N, Vissel B, Petralia RS, Wang YX, Chang K, Royle GA, Wang CY, O'Gorman S, Heinemann SF, Wenthold RJ (2003) Aberrant formation of glutamate receptor complexes in hippocampal neurons of mice lacking the GluR2 AMPA receptor subunit. J Neurosci 23:9367-9373.

Seeburg PH, Hartner J (2003) Regulation of ion channel/neurotransmitter receptor function by RNA editing. Curr Opin Neurobiol 13:279-283.

Shimshek DR, Jensen V, Celikel T, Geng Y, Schupp B, Bus T, Mack V, Marx V, Hvalby O, Seeburg PH, Sprengel R (2006) Forebrain-specific glutamate receptor B deletion impairs spatial memory but not hippocampal field long-term potentiation. J Neurosci 26:8428-8440.

Toth K, McBain CJ (1998) Afferent-specific innervation of two distinct AMPA receptor subtypes on single hippocampal interneurons. Nature neuroscience 1:572-578.

Tsien JZ, Huerta PT, Tonegawa S (1996a) The essential role of hippocampal CA1 NMDA receptor-dependent synaptic plasticity in spatial memory. Cell 87:1327-1338.

Tsien JZ, Chen DF, Gerber D, Tom C, Mercer EH, Anderson DJ, Mayford M, Kandel ER, Tonegawa S (1996b) Subregion- and cell type-restricted gene knockout in mouse brain. Cell 87:1317-1326.

Vissel B, Royle GA, Christie BR, Schiffer HH, Ghetti A, Tritto T, Perez-Otano I, Radcliffe RA, Seamans J, Sejnowski T, Wehner JM, Collins AC, O'Gorman S, Heinemann SF (2001) The role of RNA editing of kainate receptors in synaptic plasticity and seizures. Neuron 29:217-227.
