# Supplementary figures and images for: A Role for Calcium-Permeable AMPA Receptors in Synaptic Plasticity and Learning
Source: PLoS One. 2010 Sep 29;5(9):e12818. doi: 10.1371/journal.pone.0012818 (PMC2947514; doi:10.1371/journal.pone.0012818)

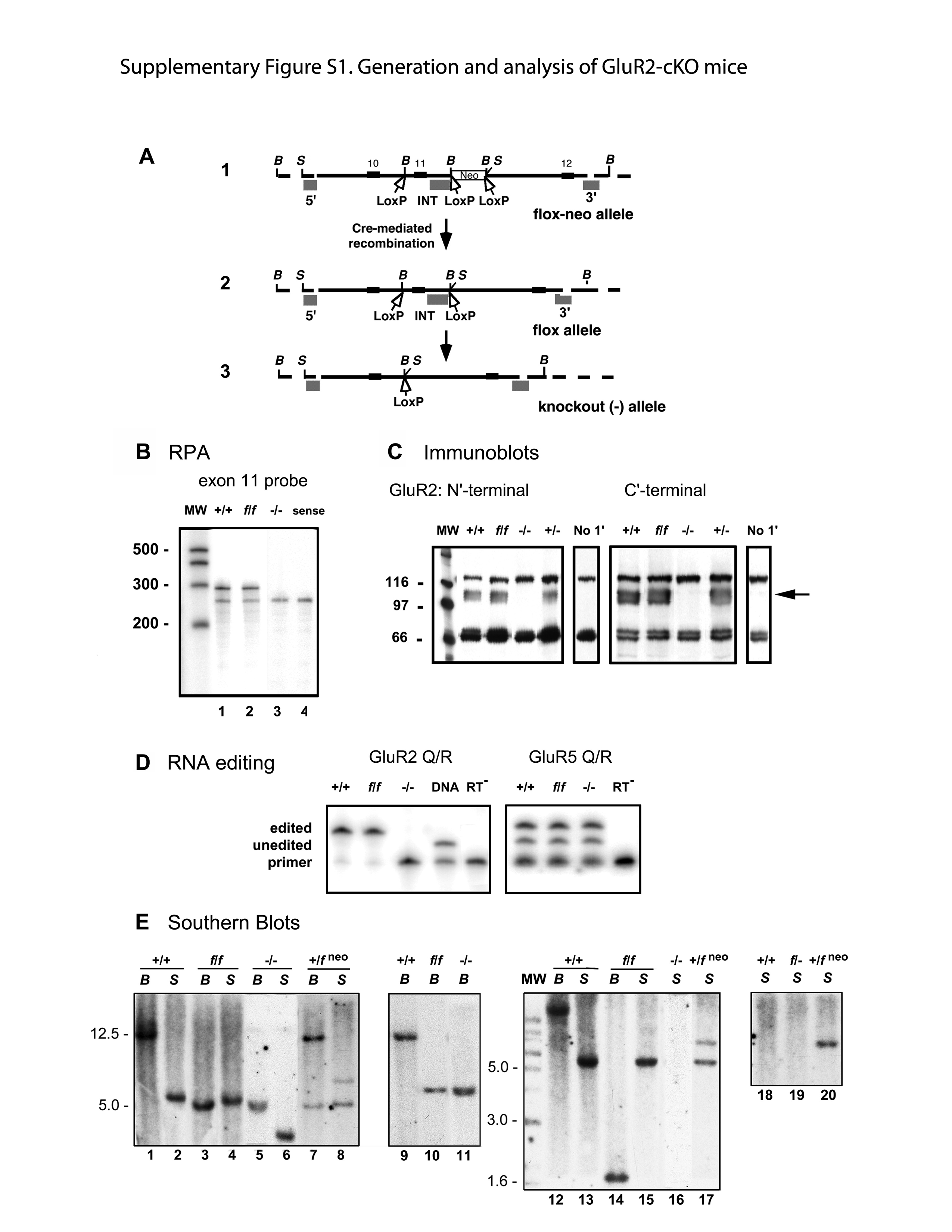

Supplement: Figure S1 — (A1) The GluR2 flox-neo allele. Shown are exons 10, 11 and 12, the loxP sites (open arrows), neomycin resistance (NeoR) gene, targeting construct (indicated by solid line), and 5′, 3′ and INT probes used for Southern blotting (gray boxes). Exon 11 encodes membrane domains 1 and 2 of the GluR2 protein, including the critical site of RNA editing (Q/R site). B = BamHI, S = SpeI. (A2) The floxed GluR2 (fGluR2) allele. Cre transfection of ES cells containing the GluR2 flox-Neo allele results in excision of the NeoR gene in some ES cells. These cells were then used to generate fGluR2 mice. (A3) The GluR2 knockout (GluR2-KO) allele. In other ES cells, transfected cre excises the exon 11-containing fragment and NeoR gene entirely from the GluR2 gene, leaving a single loxP and BamHI site in its place. These ES cells were used to generate GluR2-KO mice. (B) GluR2 RNA is expressed normally in fGluR2 but not in GluR2-KO mice. The RNAse protection assay (RPA) confirms loss of GluR2 mRNA in the GluR2-KO which was generated by cre recombinase-mediated excision of exon 11, and reveals that hippocampal GluR2 mRNA expression is normal in the WT and homozygous fGluR2 animals. The upper band (lanes 1 and 2) is the protected antisense 32P-labelled RPA probe, while the lower band (also observed in lanes 3 and 4) is an actin antisense probe included as an internal standard. The sense control probe (lane 4) is also shown. A non-RNAse-treated sample was analyzed in parallel for each probe to confirm the expected size reduction of the protected species due to removal of the 5′ and 3′ overhangs of the RPA probe (not shown). (C) Normal GluR2 protein expression in fGluR2 mice but not GluR2-KO mice. Immunoblotting was used to detect the presence of GluR2 in the hippocampus, using an antibody directed against either the N'-terminal or C'-terminal region of the GluR2 protein. 10 ug membrane protein was analyzed in each case. Note that for both antibodies, no GluR2 band is seen when the primary ant [file pone.0012818.s002.tif]

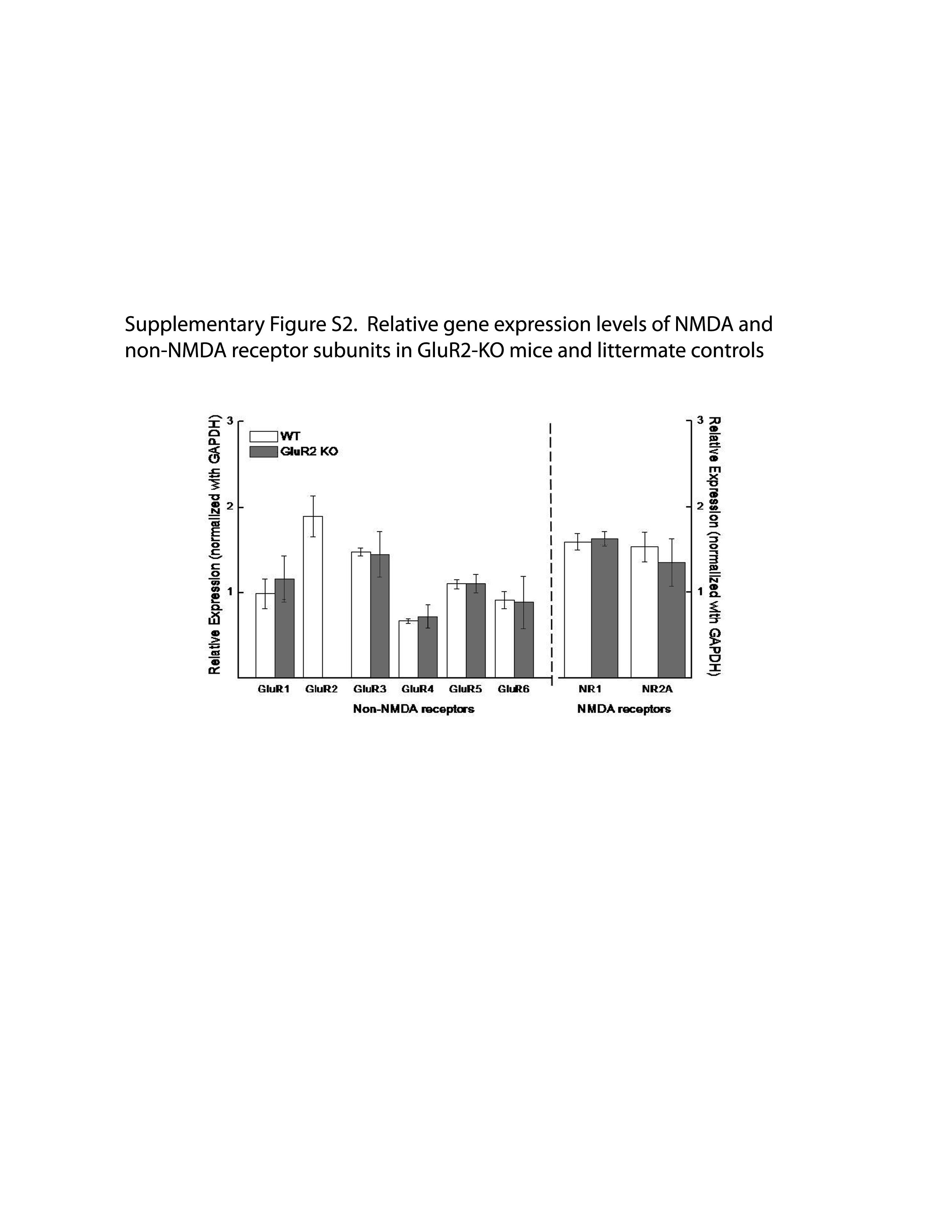

Supplement: Figure S2 — Relative gene expression of NMDA and non-NMDA receptor subunits in GluR2-KO mice (n = 5) and their littermate controls (n = 6), normalized against GAPDH. Knock-out of GluR2 did not alter gene expression of NMDA or non-NMDA receptor subunits. (0.49 MB TIF) [file pone.0012818.s003.tif]

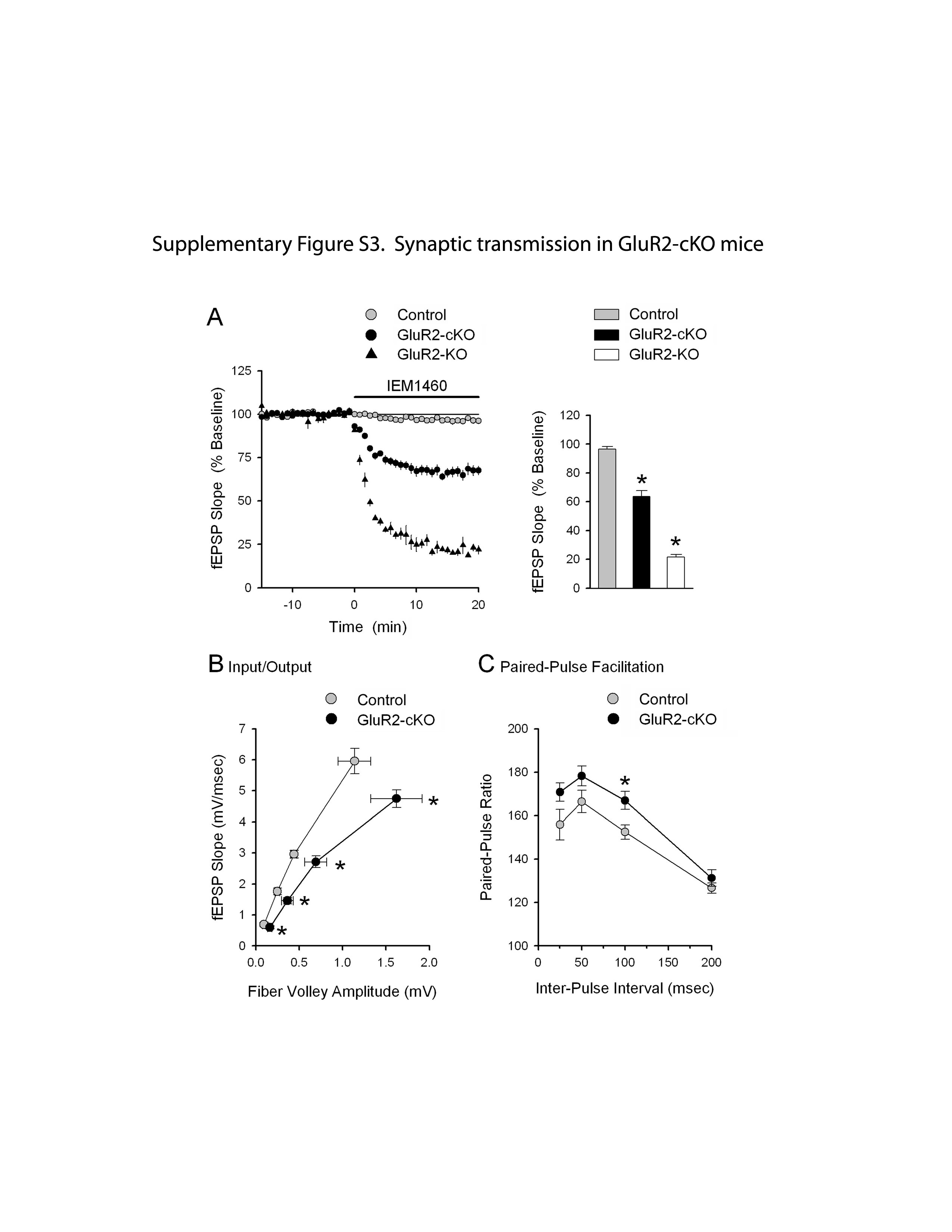

Supplement: Figure S3 — (A) The GluR2-lacking AMPAR blocker IEM1460 inhibits synaptic transmission in the CA1 region of hippocampal slices from GluR2-cKO mice (n = 3) and global GluR2 KO mice (triangles, n = 3) but has no effect on transmission in control slices (gray symbols, n = 3). Bath application of IEM1460 (100–200 Î¼M) is indicated by the bar. The histograms at right show the mean (Â±SEM) change in synaptic transmission present at the end of a 30-minute application of IEM1460 in slices from wild type (gray bar), GluR2-cKO (black bar), and global GluR2 KO mice (open bar, *p<0.001 compared to control). (B) The input/output function for basal synaptic transmission was generated by comparing fiber volley amplitudes and fEPSP slope for fEPSPs evoked using stimulation intensities corresponding to 25, 50, 75, and 100% of the maximal fEPSP amplitude. Note that the input/output function in slices from GluR2-cKO mice (black, n = 6) is shifted to the right compared to control slices (gray, n = 6). Fiber volley/EPSP slope ratios in slices from GluR2-cKO mice were significantly different from control slices at all stimulation intensities tested (* p<0.005). (C) Paired-pulsed facilitation in control and GluR2-cKO slices. Pairs of presynaptic fiber stimulation pulses were delivered with inter-pulse intervals from 25 to 200 milliseconds and the ratio was calculated as the slope of the 2nd response/1st response X 100. Although paired-pulse facilitation tends to be large in slices from GluR2-cKO mice (black, n = 5) compared to control slices (gray, n = 5), a significant enhancement was seen only at the 100 millisecond interval (*p<0.02). (0.45 MB TIF) [file pone.0012818.s004.tif]

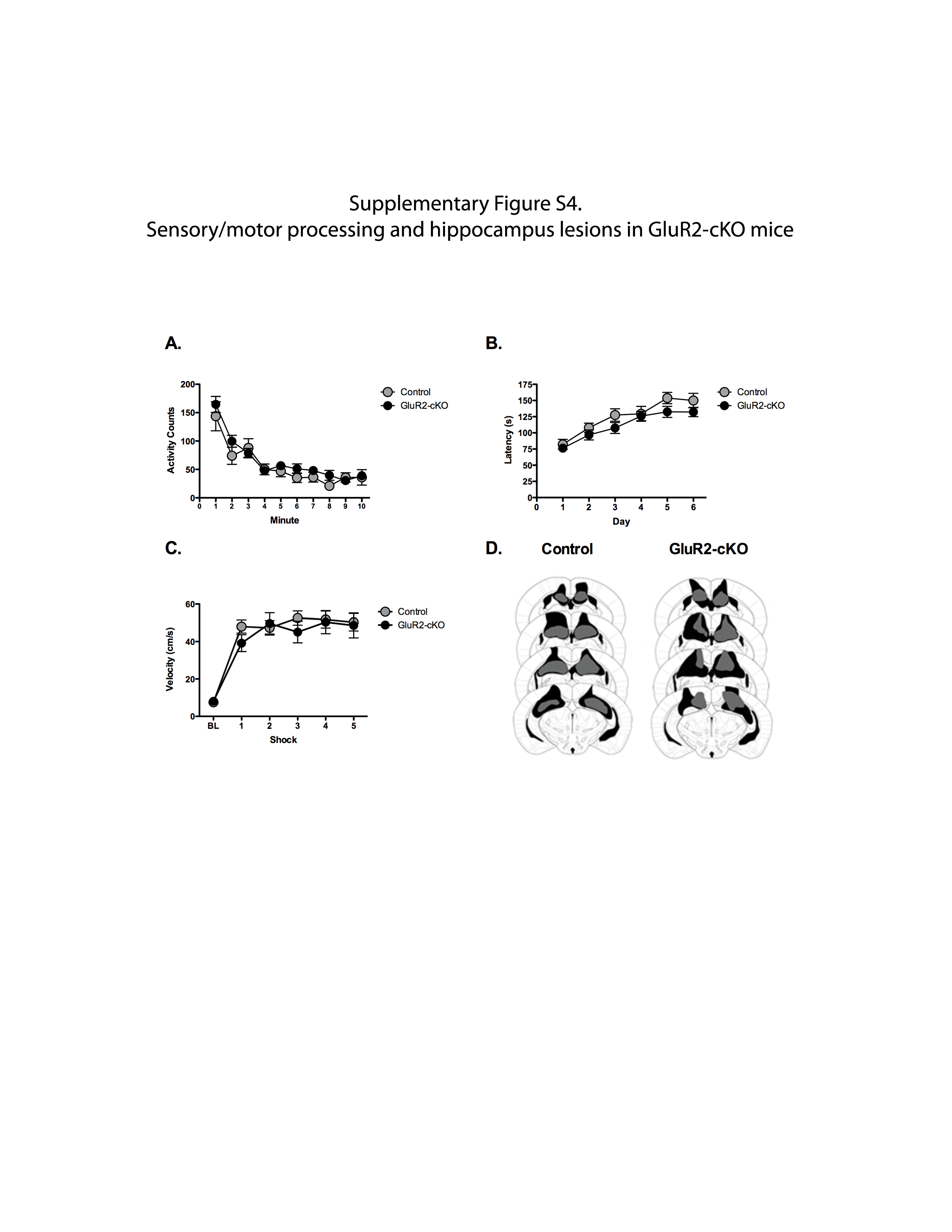

Supplement: Figure S4 — Error bars represent Â± SEM and * indicates statistical significance (p<.05). (A) Controls (n = 9) and GluR2-cKO (n = 14) showed similar levels of activity on the openfield. (B) Motor learning on the Rotarod was equivalent in controls (n = 9) and GluR2-cKO (n = 14) mice. (C) The unconditioned response to shock was examined by calculating the velocity (cm/s) of each animal during footshock. Data for the 1 (controls n = 13, GluR2-cKO n = 13) and 5 (controls n = 21, GluR2-cKO n = 12) shock groups were combined for the graph. Controls and GluR2-cKO mice showed equivalent increases in velocity during shock relative to baseline. (D) The minimum (gray) and maximum (black) extent of excitotoxic dorsal hippocampus lesions in controls (n = 6) and GluR2-cKO mice (n = 9). (0.65 MB TIF) [file pone.0012818.s005.tif]

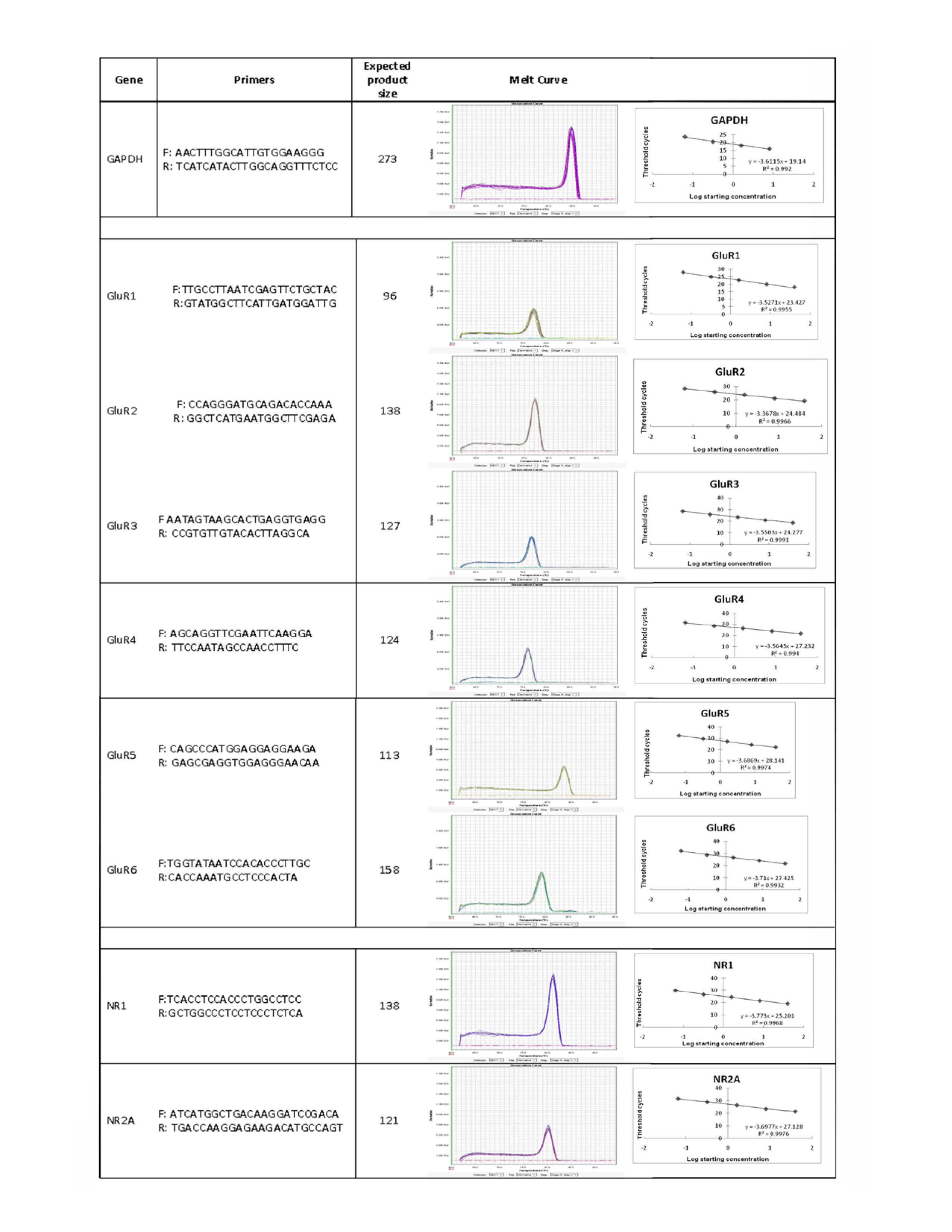

Supplement: Table S1 — Standard curves and melt curves are shown for qRT-PCR analysis of gene expression of non-NMDA receptor subunits (GluR 1, GluR2, GluR3, GluR4, GluR5, GluR6), NMDA receptor subunits (NR1, NR2A), and the calibrator gene (GAPDH). (2.20 MB TIF) [file pone.0012818.s006.tif]
